# Coacervation in Slow Motion: Kinetics of Complex Micelle Formation Induced by the Hydrolysis of an Antibiotic Prodrug

**DOI:** 10.1021/acs.molpharmaceut.4c00579

**Published:** 2024-07-16

**Authors:** Thomas D. Vogelaar, Szymon M. Szostak, Reidar Lund

**Affiliations:** † Department of Chemistry, University of Oslo, P.O. Box 1033 Blindern, Oslo NO-0315, Norway; ‡ Hylleraas Centre for Quantum Molecular Sciences, University of Oslo, Oslo NO-0315, Norway

**Keywords:** antibiotics, colistin methanesulfonate, drug delivery, mass spectrometry, small-angle X-ray scattering

## Abstract

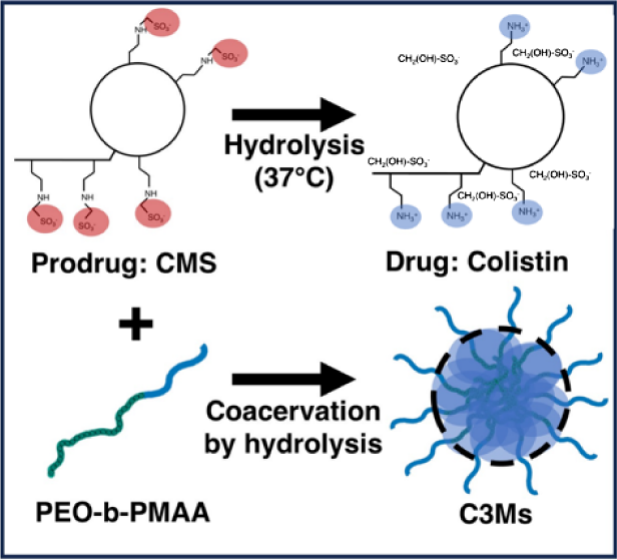

Colistin methanesulfonate (CMS) is the less-toxic prodrug of highly nephrotoxic colistin. To develop and understand highly necessary new antibiotic formulations, the hydrolysis of CMS to colistin must be better understood. Herein, with the addition of poly(ethylene oxide)-b-poly(methacrylic acid) (PEO-b-PMAA) to CMS, we show that we can follow the hydrolysis kinetics, employing small-angle X-ray scattering (SAXS) through complex coacervation. During this hydrolysis, hydroxy methanesulfonate (HMS) groups from CMS are cleaved, while the newly formed cationic amino groups complex with the anionic charge from the PMAA block. As the hydrolysis of HMS groups is slow, we can follow the complex coacervation process by the gradual formation of complex micelles containing activated antibiotics. Combining mass spectrometry (MS) with SAXS, we quantify the hydrolysis as a function of pH. Upon modeling the kinetic pathways, we found that complexation only happens after complete hydrolysis into colistin and that the process is accelerated under acidic conditions. At pH = 5.0, effective charge switching was identified as the slowest step in the CMS conversion, constituting the rate-limiting step in colistin formation.

## 1 Introduction

Over the last decades, the problems with multidrug-resistant gram-negative bacteria have been exceeding the capabilities of conventional antibiotics.^1−4^ Consequently, an increasing need for alternatives has emerged, increasing the research on antimicrobial peptides (AMP).^4−8^ One of these AMPs, colistin, from the class of polymyxins, is a cyclic lipopeptide that is highly effective against gram-negative bacterial infections.^9,10^ After reports of high neuro- and nephrotoxicity levels, colistin use is limited to last-resort treatment of bacterial infections in patients with cystic fibrosis or patients suffering from severe intestinal inflammation.^9,11^ Two different types of colistin are used in clinical settings: colistin sulfate (CS) for topical or oral treatments and colistin methanesulfonate (CMS) for aerosol or parenteral purposes.^12,13^ CMS is the inactive prodrug of CS that was created as an alternative to CS in the 1970s to reduce its toxicity.^14−16^ It is produced by sulfomethylation reactions of the five cationic α,γ-diaminobutyric acid residues of the colistin molecule.^2,14^ To go from prodrug to active drug, the methanesulfonate side groups of the prodrug are self-hydrolyzed in aqueous environments to form partially sulfomethylated derivatives, next to the fully hydrolyzed colistin, each having a different antimicrobial activity.^1,2^ Its hydrolysis in physiological conditions is found to be slow, taking up to several hours, and is characterized by the transformation from a net anionic to a cationic charge, eventually forming colistin with a charge of +5 at physiological pH. To speed up the reaction, the hydrolysis rate can be increased by an increased temperature and a reduction in pH.^12,17,18^ Even though CMS was developed to reduce the toxicity, the compound itself exhibits no antimicrobial activity. The hydrolysis of CMS into colistin creates a narrow therapeutic window that may lead to drug inefficiency or lurking acute kidney injury if the dosing of CMS and hydrolysis into colistin is not closely monitored and evaluated.^19^

The majority of research on CMS hydrolysis has focused on developing quantification techniques in vivo, particularly in animal or human plasma. These methods involve sample preparation techniques such as protein precipitation or solid phase extraction.^20^ For detection, (U)HPLC coupled with either MS/MS,^12,17,19,21−24^ UV,^24−26^ or fluorescence are used.^13,20,27^ Next to the in vivo research, a minor part of the research is done on the pharmacokinetic/pharmacodynamic (PK/PD) properties of CMS and colistin in in vitro studies.^2,24,27−30^ PK/PD models are constructed based on the in vitro hydrolysis and antimicrobial activity against relevant bacteria like *Pseudomonas aeruginosa*.^28−30^ Other in vitro research mainly focuses on the stability of CMS and CS in aqueous solutions by measuring the CMS and CS content over time, quantified by similar analytical techniques that are used in the in vivo studies.^2,22,27^ It is imperative to address the CMS hydrolysis conditions since there is a noteworthy difference between the PK/PD in vivo results and the in vitro studies. For example, Zhu et al. reported a hydrolyzed fraction of 20–25% to colistin in in vivo settings,^31^ while in in vitro, depending on the supplier, 60% to 80% of CMS can be converted to colistin after 72 h at 37 °C at pH values between 6.3 and 6.9.^1,2^ Despite extensive research on the hydrolysis of CMS, a notable knowledge gap persists. First, there are no reports of quantification methods of the intermediate sulfomethylated derivatives during hydrolysis, an absence that was also acknowledged by several authors in the field.^2,18,19,27^ Moreover, to analyze these derivatives directly, it is required to distinguish between 2^5^ = 32 structures in the hydrolysis from prodrug to drug, which is a difficult task to do accurately.^2,19^ On top of that, MS methods can potentially be unreliable due to the sulfomethylated species coeluting with CS, making thorough method validation highly necessary.^19,27^ Additionally, CMS from different suppliers was found to contain a slightly differing degree of sulfomethylation and composition.^18,25^ Lastly, there is little literature coverage on the kinetics of CMS degradation. Even though Dagla et al.^12^ reported the temperature dependency of the first-order degradation kinetics of CMS, the intermediate states were not addressed. Fundamentally filling these knowledge gaps could be crucial in the use and understanding of CMS and CS in clinical settings.

In a previous study in which we investigated CS, we showed that the cationic colistin could be complexed by a partly oppositely charged block copolymer, PEO-b-PMAA (poly(ethylene oxide)-b-poly(methacrylic acid)), forming complex coacervate core micelles (C3Ms).^32^ C3Ms generally consist of a charge-dense polyelectrolyte core containing combinations of oppositely charged polymers,^33,34^ peptides/proteins,^35−37^ DNA,^38,39^ or drugs.^40−43^ The C3M cores are typically sterically stabilized through the presence of polymer brushes conjugated to the charged species present in the core. These polymer brushes are usually composed of charge-neutral blocks like PEO, ensuring effective entropic stabilization.^32,33,40,41,44,45^ To characterize C3Ms, their size, shape, and structure are often analyzed through scattering techniques.^32,46^ However, most work has been focussed on static properties and less emphasis has been made on the formation and exchange kinetics, even though the dynamic properties are essential to for the stability and structural control.^47^ The formation kinetics of C3Ms typically occur in millisecond range, making formation kinetics challenging to resolve.^33,48,49^ To elucidate the formation kinetics of complex coacervation, fast mixing techniques are often combined with turbidity, X-ray, or light scattering measurements.^50^ As an alternative to fast mixing, chemical clock reactions can be used to control the timing of the start of the complexation, although they do not decelerate its formation kinetics.^51−53^

In this work, we combine SAXS, allowing nanostructural resolution, with MS and determine the hydrolysis profile of CMS reflected in the release of hydroxy methanesulfonate (HMS). The associated charge reversal leads to the growth of coacervate micelles in the presence of PEO-b-PMAA block copolymers. The results indicate that the rate-limiting step of CMS hydrolysis is the net charge reversal (from negative to positive charge), whereas the rate constant of the first CMS hydrolysis step is significantly larger and concentration-independent. Additionally, we find that C3Ms can be formed only in the presence of fully hydrolyzed CMS in the form of colistin. Finally, we present a novel way to study the decelerated formation kinetics of well-studied colistin-C3Ms, slowed down by the hydrolysis of CMS, resulting in a shift in the time scale from milliseconds to hours.

## 2 Experimental Section

### 2.1 Materials

All chemicals were purchased and used without further purification. Colistin methanesulfonate (CMS, PHR1737), colistin sulfate (CS, PHR1605), maleic acid, citric acid, trisodium citrate, Trizma base, Trizma hydrochloride, sodium hydroxide (NaOH), hydrochloric acid (HCl), and methanesulfonic acid were purchased at Sigma-Aldrich/Merck. Poly(ethylene oxide)-b-poly(methacrylic acid) (PEO_45_-b-PMAA_41_, 2-b-3.5 kDa, PDI = 1.20) was purchased at Polymer Source Inc.

### 2.2 Buffer Preparation

For the experiments, six different buffers (between pH values of 5.0 and 8.7) were prepared. Since buffering agents only have buffering capacities in small pH regions, three different buffers were used: citrate, maleate, and TRIS. The buffers were prepared at 0.05 M at 37 °C; citrate at pH = 5.0; maleate at pH = 6.0 and pH = 7.0; and TRIS at pH = 7.4 (physiological pH), pH = 8.0, and pH = 8.7.

### 2.3 Standard Sample Preparation Method

The PEO-b-PMAA polymer was dissolved in each buffer to obtain a stock solution of 10 mg/mL. Previously, it was found that charge-matching conditions (equal charges of PMAA and colistin) led to the most stable complex core coacervate micelles (C3Ms).^32^ With the assumption that all CMS would be hydrolyzed to colistin (from an effective charge of −5 to +5), a charge ratio (*f*_+_) of 0.5 (equal charges in the final state) was used. CMS was dissolved in the corresponding buffers and then properly mixed with PEO-b-PMAA solution under charge-matching conditions (PEO-b-PMAA:CMS 1:2.6 mass ratio) to a final concentration of 10.0 mg/mL (7.2 mg/mL CMS), 7.5 mg/mL (5.4 mg/mL CMS), 5.0 mg/mL (3.6 mg/mL CMS), or 2.5 mg/mL (1.8 mg/mL CMS). These well-mixed solutions were then incubated at 37 °C using a water bath or sample heater, indicating the start of the hydrolysis (*t* = 0 h).

### 2.4 Surface Tension Measurements

Surface tension measurements were performed using a pendant drop tensiometry setup from ramè-hart instruments. Images of the drops were captured with a CCD camera, and surface tension was determined using DROPimage Advanced v 3.19.12.0. For each measurement point, 100 frames were captured and the surface tension was determined and averaged. To determine the CMC, two regions were distinguished between 0.0 and 5.0 mg/mL of total concentration. The transition point corresponds to the CMC.

### 2.5 Small-Angle X-Ray Scattering

Small-angle X-ray scattering (SAXS) experiments were performed on two different instruments at the ESRF synchrotron in Grenoble, France, and on the in-house SAXS at the University of Oslo, Norway.

#### 2.5.1 Synchrotron SAXS

The static SAXS profiles were measured at 37 °C using the BioSAXS beamline BM29^54^ at the European Synchrotron Radiation Facility (ESRF) in Grenoble, France. The automated sample changer was put to load 50 μL for every sample into a quart glass capillary of a diameter of 1 mm. Ten scattering frames of 1.0 s each were detected on the Pilatus 3 × 2 M detector, using an energy of 12.5 keV and a sample–detector distance of 2.81 m, measuring a *q*-range of roughly 0.007–0.55 Å^–1^. The background sample (the corresponding buffers) was measured before and after each sample measurement, and the capillary was cleaned between every measurement. Water was used as a primary standard to scale the data to the absolute intensity. Every frame was assessed for radiation damage, followed by averaging, buffer subtraction, and binning (from 1000 points to 280), resulting in the final scattering curves presented in this paper.

#### 2.5.2 In-House SAXS

For the time-dependent SAXS measurements, a Bruker NanoStar diffractometer, located in the RECX instrument lab at the University of Oslo, Norway, was used. The newly mixed sample of CMS and PEO-b-PMAA was injected and measured every hour at 37 °C for 72 h. The measurement time was 3600 s, thereby measuring an average scattering pattern inside that hour. The background sample (the corresponding buffers) was measured and subtracted. Water was used as a primary standard to scale the data to the absolute intensities. Lastly, the scattering patterns were binned to 200 points.

### 2.6 Mass Spectrometry (MS)

CMS was dissolved in a buffer, containing the internal standard, methanesulfonic acid (1 mg/ml), at three different concentrations (1.8, 3.6, or 7.2 mg/mL) and heated in a water bath at 37 °C. PEO-b-PMAA was not added to not contaminate the MS. Experiments were performed on the Bruker maXis II ETD MS instrument at the University of Oslo. Samples of 10 μL were taken, diluted with Milli-Q water, and then injected. The samples at pH = 5.0 were ionized by electrospray in negative mode with a capillary voltage of 3500 V and an end plate offset of −500 V; nebulized at 0.5 bar; and dry heated at 200 °C with a dry gas flow of 4 L/min. Ions in the range of 50 to 500 *m*/*z* were detected. The hydrolysis was determined by the comparison of the intensity of the [CH_2_(OH)SO_3_]^−^ hydroxy methanesulfonate (HMS) ion (110.9758 *m*/*z*) signal and scaled with the intensity of the internal standard [CH_3_SO_3_]^−^ ion (94.9808 *m*/*z*) signal. To follow the progress of the reaction of CMS, these intensities were divided by the normalized intensity of the final state at the corresponding pH values (heated at 37 °C for 72 h).

## 3 Results and Discussion

**Figure 1:**
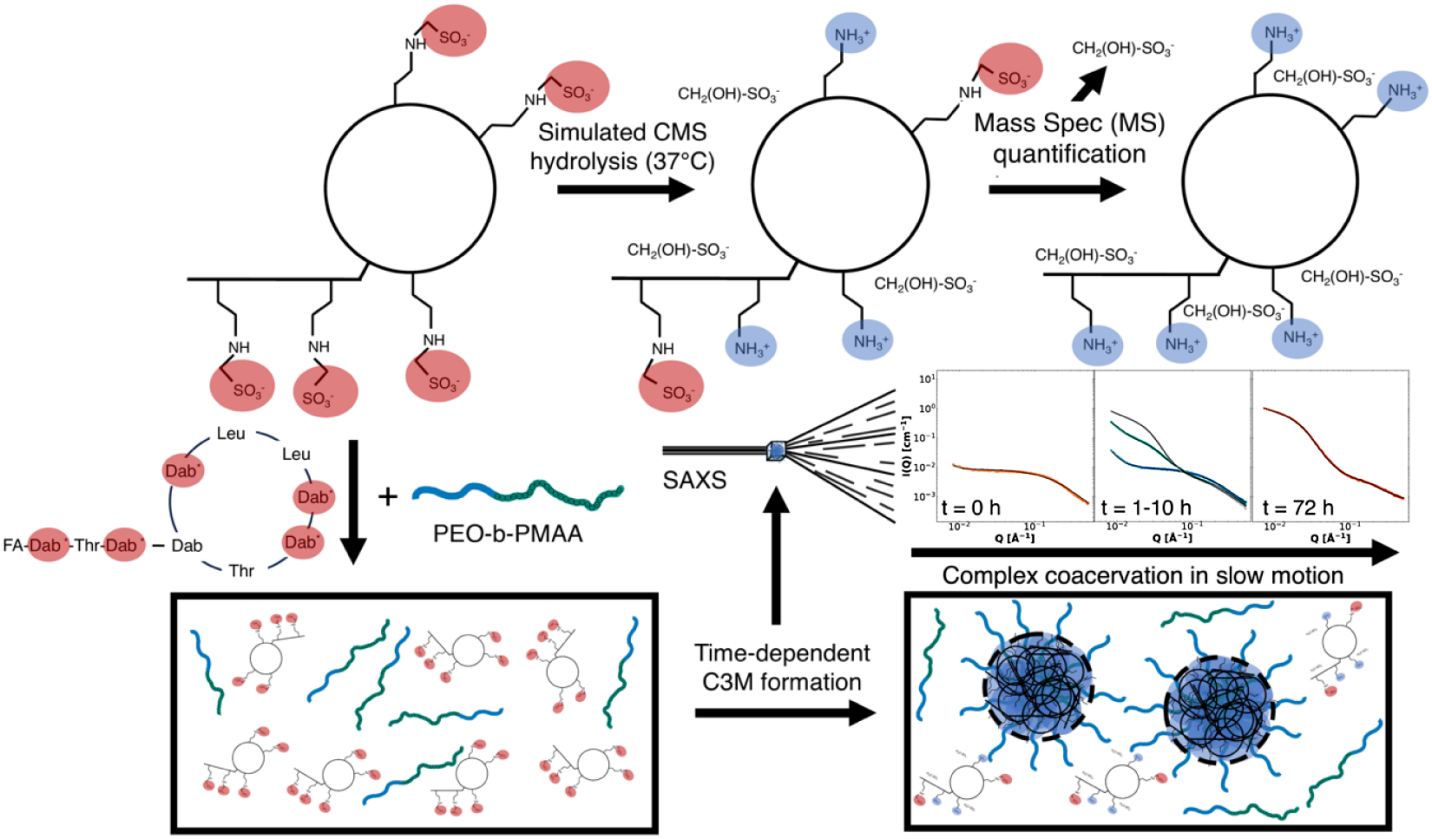
Schematic representation of the conducted research on CMS (structural formula in Figure S1A) hydrolysis in aqueous solutions. The sulfomethylated groups of α,γ-diamino butyric acid (Dab) side groups (indicated by an asterisk and in red) are hydrolyzed to cationic amine groups (blue) (top row). When the block copolymer PEO-b-PMAA (structural formula in Figure S1B) is added, the hydrolysis can be followed using SAXS since PEO-b-PMAA forms complex core coacervate micelles (C3Ms) with the newly formed colistin. During the hydrolysis of CMS, HMS molecules are released, which are quantified employing MS. Since the hydrolysis of CMS is the rate-limiting factor in micellization, the C3M formation can be followed in slow motion using SAXS modeling to map out the formation of complex coacervates based on their composition, size, and structure.

To quantify CMS hydrolysis experimentally, we developed two approaches. First, we studied the hydrolysis at 37 °C in the presence of PEO-b-PMAA using SAXS. As the CMS hydrolyzes and reverses its charge, complexation with the partly anionic polymer is expected, leading to an increase in the intensity as the nanoparticles grow. Additionally, the time-resolved SAXS data provide access to the overall nanostructure including shape and size elucidation. Second, the hydrolysis was determined by monitoring the hydroxy methanesulfonate (HMS) release. A schematic representation of the experimental design is presented in Figure 1.

### 3.1 Following CMS Hydrolysis through Polymer Complexation

**Figure 2:**
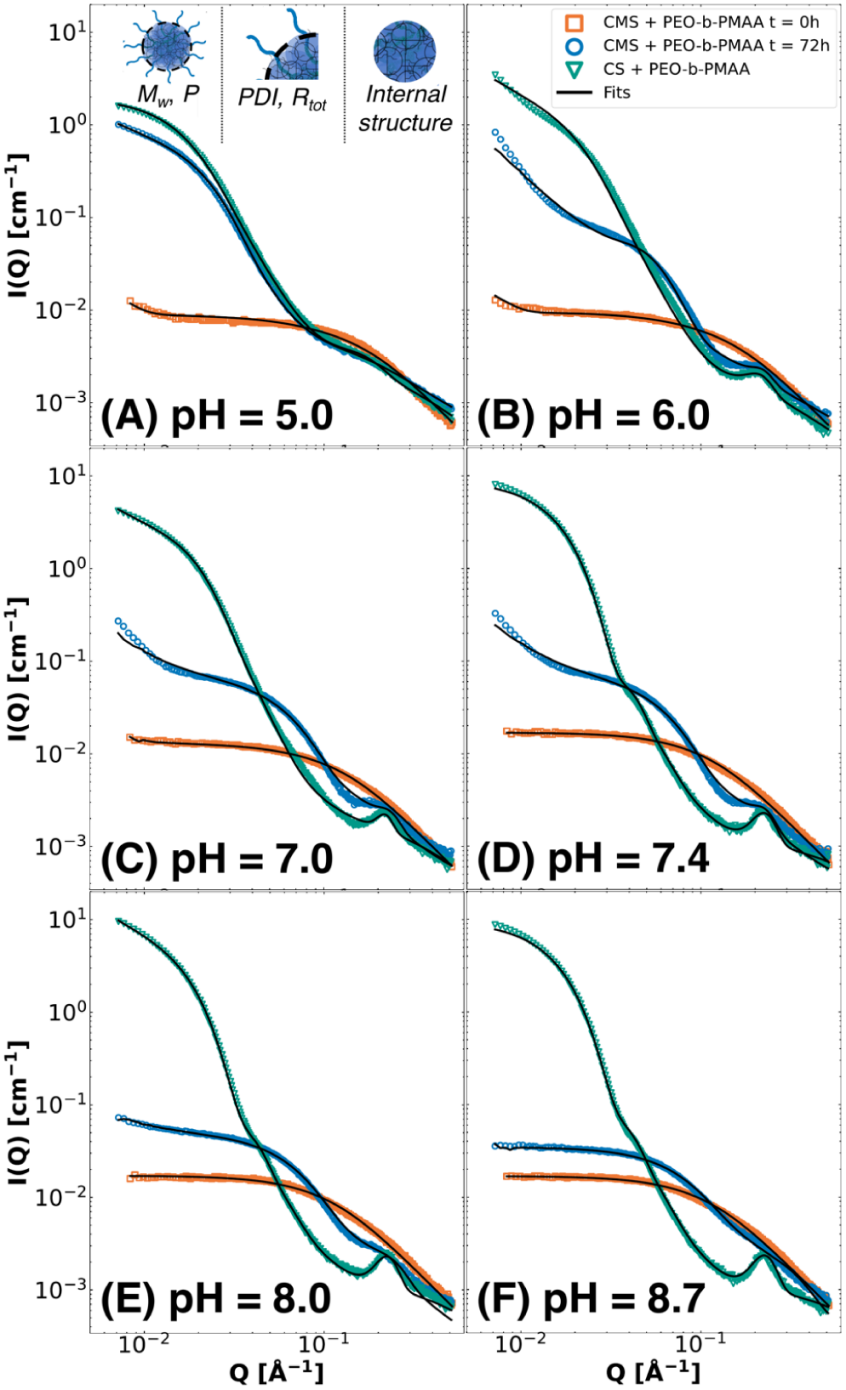
Synchrotron SAXS patterns of the pH dependence of the coacervation and hydrolysis process of CMS with PEO-b-PMAA at 5.0 mg/mL, including fits from the CS/CMS complex coacervate model. In the top left corner, the relation between the structural characteristics of C3Ms and their corresponding SAXS data is illustrated. The starting point of CMS + PEO-b-PMAA (orange squares), the final state of CMS + PEO-b-PMAA (blue circles), and the “control” of CS C3Ms (green triangles) are indicated for pH = 5.0 (A), pH = 6.0 (B), pH = 7.0 (C), pH = 7.4 (D), pH = 8.0 (E), and pH = 8.7 (F). The CS + PEO-b-PMAA coacervates have matching concentrations considering the maximum conversion of CMS into colistin.

It is well documented that the hydrolysis of CMS is highly pH-dependent.^12,17^ Moreover, the effect of pH on (the final stages of) the hydrolysis of CMS needs to be assessed before performing more detailed time-resolved kinetic measurements. The start and final states of CMS hydrolysis were analyzed (incubation for 72 h at 37 °C)^2^ at six different pH values (pH = 5.0, 6.0, 7.0, 7.4, 8.0, and 8.7) with PEO-b-PMAA as the complexing agent using SAXS, and compared to the previously extensively characterized colistin C3Ms (Figure 2), in which CS was complexed directly as a control.^32^ Upon complete hydrolysis, CMS loses all of its five methanesulfonate groups in the form of HMS and is converted into colistin. As the cationic groups present on the hydrolysis products can readily form complexes with the partly anionic PEO-b-PMAA, this leads to an increase in the molecular weight of the micelles, which is reflected in the measured increase in scattered intensity. Since CMS cannot form coacervate micelles with like-charged polymers, the growth can be related to the hydrolysis of HMS from CMS. However, it is unclear how many charges are necessary for the hydrolysis products to effectively complex with the partly anionic PEO-b-PMAA. To resolve the composition, shape, size, and structure,^46^ all the data sets were analyzed on an absolute scale using a model for spherical polydisperse micellar structures with graded interfaces,^32,33,55−58^ taking into account the mass balance of possible structures (micelles, free polymers/peptides). This CS/CMS complex coacervate model consists of three separate contributions: complex coacervate scattering; free (noncomplexed) polymer and colistin, CMS; and an additional structure factor describing the internal structure (charge correlations between “blobs”) between polyelectrolytes in the core, *S*(*Q*)_internal_. The complex coacervate scattering (*I*_Coa_(*Q*)) is described by a fuzzy-sphere form factor,^32,55,56^ including a structure factor for cluster formation (aggregates), *S*(*Q*)_cluster_,^59^ and a blob contribution, blob(*Q*).^32,33,57,58,60^ The free scattering contributions (*I*_Poly,free_(*Q*), *I*_Col,free_(*Q*), and *I*_CMS_(*Q*)) make use of the Debye scattering form factor for polyelectrolytes.^61^ The complete scattering function is described in eq 1.
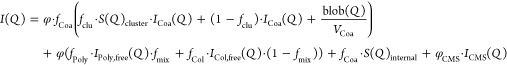
1

where φ is the volume fraction, *f*_Coa_ is the fraction of coacervates, *f*_clu_ is the fraction of coacervates that are forming clusters, *V*_Coa_ is the volume of one complex coacervate, *f*_Poly_ is the free fraction of the polymer, *f*_Col_ is the free fraction of colistin, *f*_mix_ is the molar fraction of the polymer in the aqueous phase surrounding the complex coacervates, and ϕ_CMS_ is the volume fraction of CMS. ϕ_CMS_ is calculated based on the conversion rate to colistin, obtained by the kinetic model and the maximum hydrolysis described later in this section. A more detailed explanation of the CS/CMS complex coacervate model can be found in the Supporting Information. Based on least-squares fit routines, several parameters could be fitted to the data and were therefore kept free: the radius of the core, including its density distribution and polydispersity (*R*_in_, *σ*_in_, PDI), the free fraction of colistin (*f*_Col_), and the total aggregation number (*P*). For the systems showing a tendency to cluster, manifested as an upturn at low *Q*, an additional structure factor for *N*_clu_ randomly connected C3Ms at a distance *D*_dist_ set as twice the micellar radius. To describe the scattering patterns at high *Q* values, a blob scattering term, i.e., a contribution from an internal polyelectrolyte network, was included characterized by the fraction (*f*_blob_) and blob correlation length (ξ). Furthermore, to account for blob charge correlations,^32,62^ we also included a Gaussian peak function described by the width (*W*) correlation peak position (*Q*_local_).

The pH value is negatively correlated with the hydrolysis maximum intensity of CMS, while the colistin complex coacervates reach a higher intensity value at higher pH values, including a more pronounced structure factor peak at high Q (Figure 2). Based on the features observed in the scattering patterns at pH = 5.0, the hydrolyzed CMS seems to be forming similar micellar complex coacervates (C3Ms) as with CS. Upon every increase in pH, the CMS hydrolysis product, complexed by the polymer, reached a lower maximum intensity, while simultaneously, the C3Ms reached a higher intensity at increasing pH values, suggesting acid catalysis and better micellization conditions, respectively. These observations are corroborated by the fits with the CS/CMS complex coacervate model (eq 1, fitting parameters in Tables S1 and S2). At CMS hydrolysis at pH = 5.0, we found the C3Ms with the highest molecular weight (0.81 × 10^6^ Da) and size (total radius = 11.0 nm) but also the highest polydispersity (25%) (Table S1). The CMS hydrolyzes to colistin to the highest extent at both pH = 5.0 and 6.0 and this fraction decreases upon increments of the pH. Nevertheless, the formation of C3Ms at pH = 5.0 and pH = 6.0 also seems the most challenging, most likely because of the decreased polymer charge, since the p*K*_a_ of PEO-b-PMAA is reported to be between 4 and 5 (Table S2).^63^

**Figure 3:**
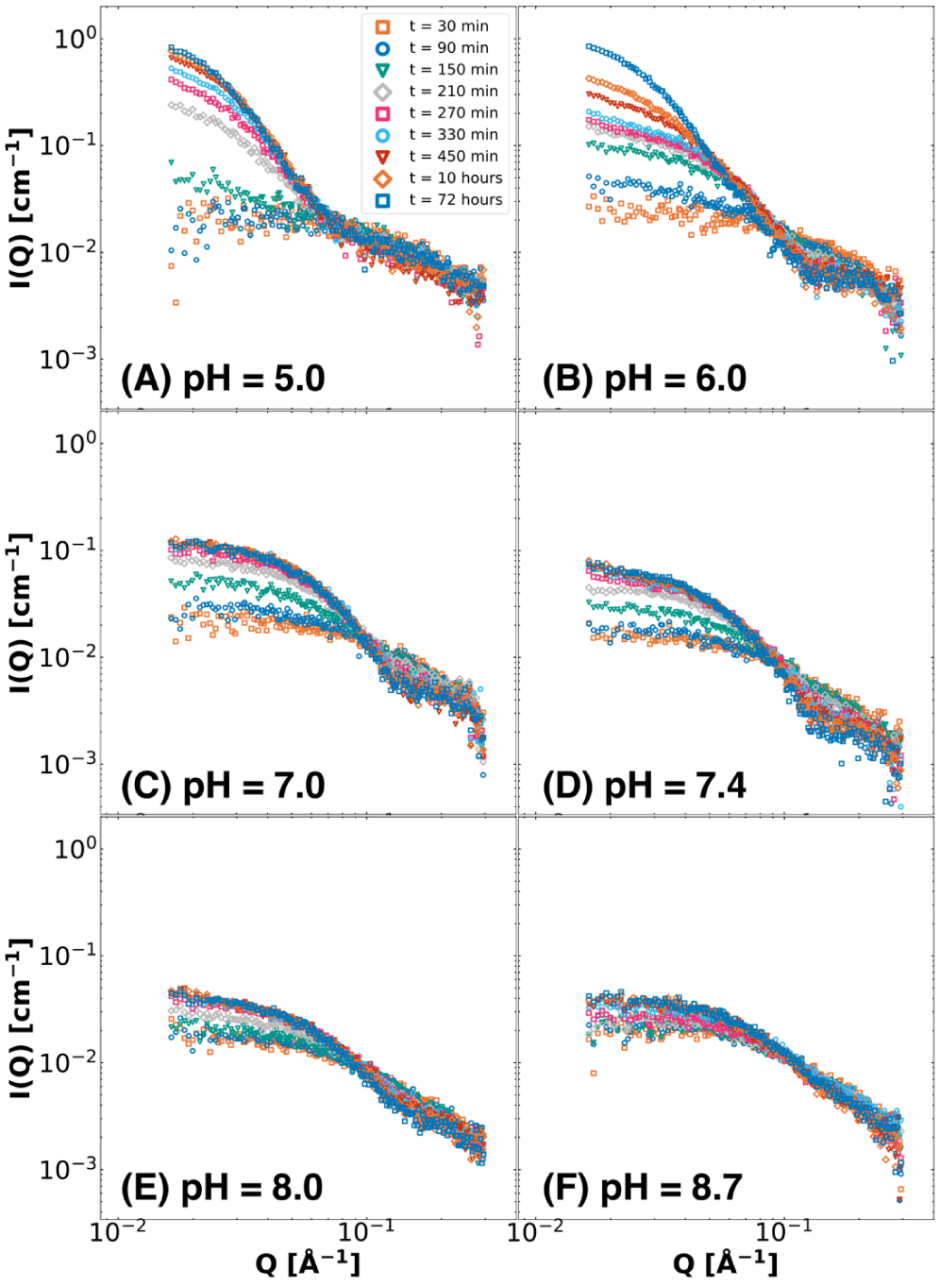
SAXS pattern representation of the pH dependence of the coacervation process over time of CMS with PEO-b-PMAA at a total concentration of 5.0 mg/mL. Coacervation over time was measured at pH = 5.0 (A), pH = 6.0 (B), pH = 7.0 (C), pH = 7.4 (D), pH = 8.0 (E), and pH = 8.7 (F) for 72 h. The same graph window was used for all plots to visualize the changes in scattering intensity between the different pH values in the clearest manner. Since the *Q*-range is smaller for the in-house SAXS, the upturns at low Q, seen in Figure 2, are less visible.

We found a clear dependence between PDI and *M*_w_ for CS C3Ms with differing pH values. We roughly observe a 10% point increase in PDI when PEO-b-PMAA is closer to the p*K*_a_ (28% at pH 5.0 and ≈16–17% at pH ≥ 7.4), as well as a factor 3 difference in *M*_w_ (1.1 × 10^6^ Da at pH = 5.0 and pH = 6.0, while *M*_w_ ≈ 3 × 10^6^ Da at pH ≥ 7.4). Additionally, we noticed an effect of the complex coacervation itself on the formation of the complexes at pH = 5.0 by comparing C3Ms formed in the presence of PEO-b-PMAA during hydrolysis versus those formed without the presence of PEO-b-PMAA, followed by the addition of PEO-b-PMAA at the end of hydrolysis (Figure S2). Even though the hydrolysis quantity seems similar, the C3M structures are different (Table S3), indicating the importance of kinetically arrested states in this complex coacervation process. Moreover, formation of C3Ms slowly during hydrolysis is the rate-limiting step that results in much smaller C3Ms (total radius of 11.0 nm versus 16.1 nm and an *M*_w_ of 0.8 × 10^6^ Da versus 2.1 × 10^6^ Da), while the other fitting parameters, e.g., the free fractions of polymer and colistin, which are modeled separately, and the polydispersity are similar. Subsequently, since the hydrolysis quantity is similar, we assume that PEO-b-PMAA does not influence the hydrolysis itself significantly. To determine the kinetics of C3M formation at these different pH values and to visualize the intermediate steps of the CMS hydrolysis and complex coacervation, we collected scattering measurements every hour for 72 h using an in-house SAXS instrument (Figure 3).

As shown in Figure 3, the kinetics of complex coacervation formation induced by CMS hydrolysis can be monitored (Figure 3). The end state is almost reached at *t* = 10 h at all pH values apart from pH = 6.0, in which the scattering intensity is significantly higher after a longer time, potentially caused by large aggregation and instability, which is indicated by its white-turbid appearance after hydrolysis (Figure 3B). Initially, the polymer and CMS do not interact with each other, and the intensity corresponds to a sum of the CMS and polymer chain scattering indicated by weak *Q*^–2^ dependence at high *Q*. This is related to the critical micelle concentration (CMC) of the system that has to be overcome to form micelles^32,40^ At around 150–270 min, it is apparent that micellar structures start to form, for 5.0 ≤ pH ≤ 7.4, as the slope in medium *Q* starts to increase, while the high *Q* scattering decreases, resembling the formation of spherical C3Ms. This micelle formation is a direct result of the CMS hydrolysis to cationic colistin, which can form complexes with the anionic PMAA blocks due to electrostatic attraction. Since the time-resolved measurements had to be acquired using in-house SAXS, we have limitations in the possibilities for accurate application of complex fitting models.

**Figure 4:**
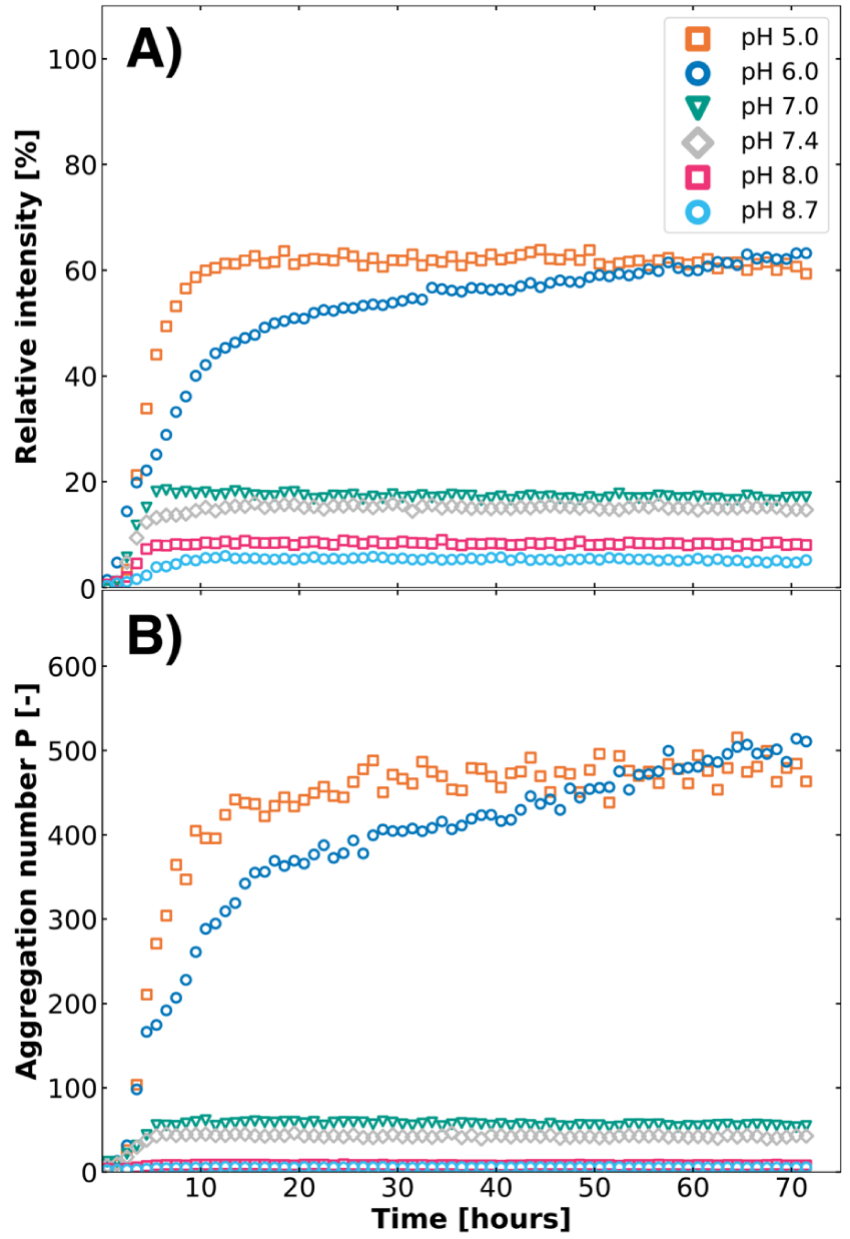
Summed scattering intensity between *Q* = 0.017 and *Q* = 0.080 Å^–1^ divided by the summed scattering intensity over the same range of C3Ms at a total concentration of 5.0 mg/mL at the corresponding pH over time (A). The aggregation number *P*, calculated by Guinier analysis, was over time for each corresponding pH (B). These relative intensities and aggregation numbers are estimates for the hydrolysis reaction of CMS, based on the formation of cationic charges for each corresponding pH value at pH = 5.0 (orange squares), pH = 6.0 (dark blue circles), pH = 7.0 (green triangles), pH = 7.4 (gray rhombuses), pH = 8.0 (pink squares), and pH = 8.7 (light blue circles).

Therefore, to gain better insights into the quantification of hydrolysis kinetics and compare pH values to each other, we summed the intensity between *Q* = 0.017 Å^–1^ and *Q* = 0.080 Å^–1^ at every measurement (Figure 3) and divided it by the maximum obtainable intensity from the control samples of CS C3Ms (Figure 2, green triangles), scaled by the number of points and corrected for *t* = 0 measurements for all pH values (eq 2). This relative intensity was plotted against time for the different pH values to observe the pH effect directly (Figure 4A). Additionally, in parallel, we performed a Guinier analysis (simultaneous fits from the lowest *Q* until *Q* = 0.02 Å^–1^ to obtain *I*(0)) to obtain estimates for the molecular weight (*M*_w_) and therefore the mean aggregation number (*P*) (eq 3) of the formed micelles to the integral intensity method (Figure 4B). By definition of the relative intensity in these ways, the hydrolysis rate and kinetic formation can be quantified, as these parameters are interdependent. Moreover, simple and accessible CMS hydrolysis quantification is possible on every SAXS, without the need for complex fit models.
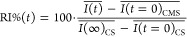
2
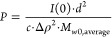
3

where  are the integral intensities of the states of CMS hydrolysis at a time point *t*, *t* = 0, the final state of CS C3Ms (∞), and the added scattering of these CS C3Ms without mixing (*t* = 0); *I*(0) is the extrapolated intensity at *Q* = 0; *d* is the average density of the system; *c* is the total concentration; Δρ is the scattering length density contrast; and *M*_w,average_ is the average molecular weight of CMS and polymer, scaled to charge matching complex coacervates.

By monitoring the increase in the SAXS intensity with the growth of the C3Ms, we see that the formation kinetics of the complexation of PEO-b-PMAA with the CMS hydrolysis derivatives can be determined (Figure 4). Employing the relative intensity method and Guinier analysis gives similar patterns in the formation of complex coacervates at pH 5.0 and 6.0 but also shows deviations. These deviations are especially pronounced at pH values of 7.0 and higher, resulting in higher variability and noise (due to lower scattering intensities) and an overestimation of the hydrolysis quantification. This effect would even be more pronounced considering the scaling factor involved in the relative intensity method. However, using both methods, the result of the pH value on the final hydrolysis state is apparent, and maximally 63% of the relative intensity could be reached at lower pH values with an aggregation number of around 480 while at physiological pH, a relative intensity around 16% was reached with an aggregation number of around 60 (Figure 4). All except pH = 6.0 reached a plateau after around 10 h, likely because of continuing aggregation and destabilization of the system, as mentioned previously. To elucidate the states of the cationic species responsible for the observed intensity growth and conduct a comprehensive compositional analysis of CMS and its hydrolysis products, we opted for pH = 5.0, as it ensures system stability and yields a higher CMS hydrolysis, especially important for employing and combining MS and synchrotron SAXS techniques.

### 3.2 Kinetic Modeling of CMS Hydrolysis

**Figure 5:**
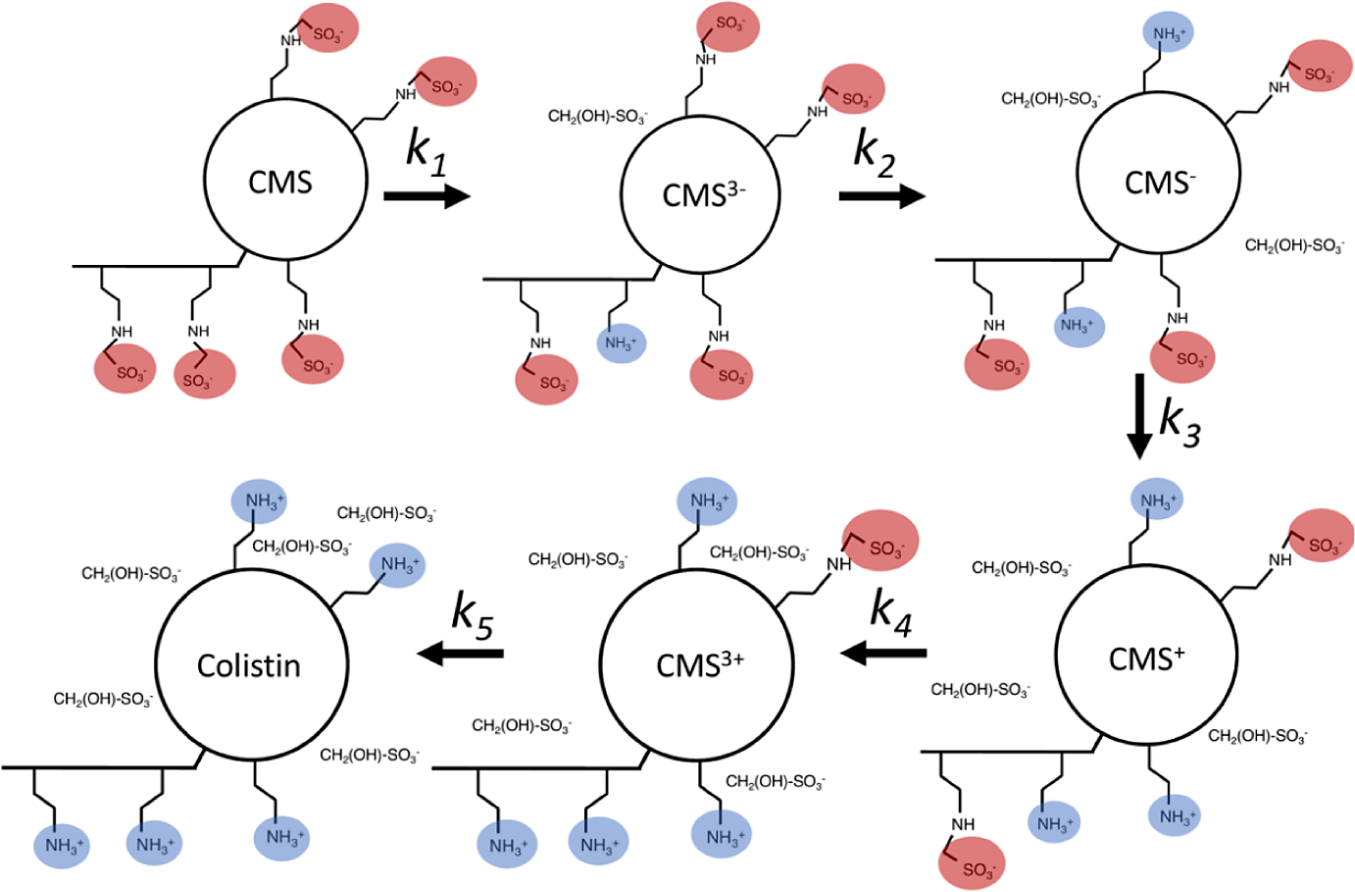
Illustration of the simplified hydrolysis pattern of CMS to colistin, in every step releasing hydroxy methanesulfonate (HMS). The assumption that the positioning of methanesulfonate and amine groups is random is made here, focusing on the differently charged species during this hydrolysis process.

To obtain a more comprehensive understanding of the kinetics, the hydrolysis of complex micelle structures (CMS) was further quantified based on the release of hydroxy methanesulfonate (CH_2_(OH)SO_3_^–^, HMS) ions. This compound represents the hydrolysis product of the preferred oxidative state of the methanesulfonate group and was analyzed by mass spectrometry (MS). The HMS intensity was then normalized using methanesulfonic acid (CH_3_SO_3_H) as an internal standard. The simplified hydrolysis process of CMS is illustrated in Figure 5, where the positioning of the remaining methanesulfonate groups is assumed to be random.

**Figure 6:**
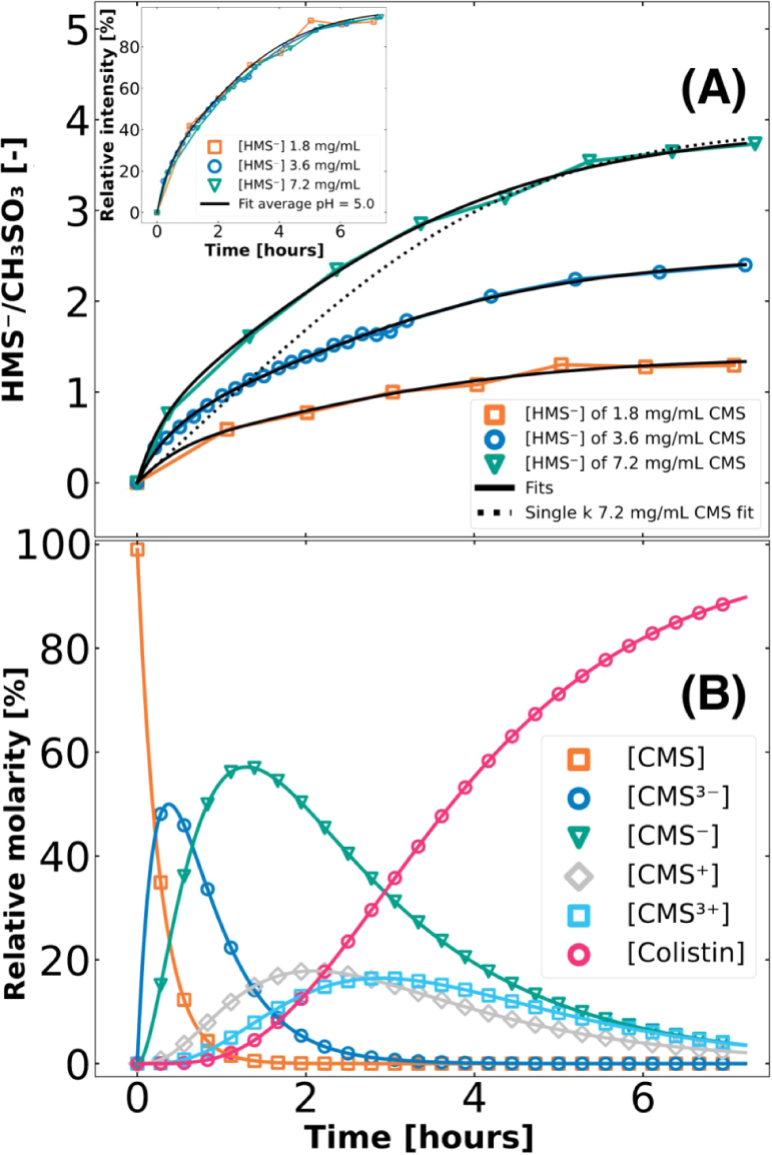
MS HMS hydrolysis data at three different concentrations of CMS: 1.8 mg/mL (orange squares), 3.6 mg/mL (blue circles), and 7.2 mg/mL (green triangles), including kinetic model fits (black lines), using five different first-order kinetic rate constants describing simplified reaction steps in CMS hydrolysis (A), including concentration-independency (inset). A single kinetic rate constant was shown to not describe the data (dotted black line). Based on the averaging of the kinetic rate constants, from the fits in (B), the kinetic profile of all versions of CMS (CMS (orange squares), CMS^3–^ (blue circles), CMS^–^ (green triangle), CMS^+^ (gray rhombuses), CMS^3+^ (light blue squares), and colistin (pink circles) could be calculated to give a generic kinetic model with relative molarities of CMS hydrolysis at pH = 5.0 (B).

Interestingly, a quick fit assuming first-order^12^ kinetics with a single rate constant did not describe the data (Figure 6A). To obtain a better understanding of the hydrolysis, we created a kinetic model involving five reaction steps, where we assume that the rate constant is only a function that depends on the number of remaining methanesulfonate groups, i.e., it is independent of the exact site (Figure 5). Based on previous literature,^1,2,12^ we assume that there is a certain fraction of CMS that is not hydrolyzed, probably due to a dynamic equilibrium that depends on the conditions (temperature and pH). The kinetic model is summarized in eq 4 (details in Supporting Information). With the relation between the kinetic model and HMS formation (eq 5), we fitted the equation to the MS data from [HMS^–^] concentrations using least-squares fitting routines for three concentrations (Figure 6A). It was found that the hydrolysis profile of HMS is concentration-independent after normalization (inset in Figure 6A). To obtain well and unique fits for those three sets of MS data, five different (first order) kinetic rate constants (eq 5) were taken as input parameters, *k*_1_, *k*_2_, *k*_3_, *k*_4_, and *k*_5_. Trial and error of the coupling of several combinations of kinetic rate constants was imposed, yet proved to be ineffective, leaving all kinetic rate constants as independent parameters. Since the formation kinetics of HMS were found to be concentration-independent, we averaged the three kinetic model fits (separate fits in Table S4) and presented them in Table 1. Subsequently, the average kinetic rate constants were applied to eq 5, resulting in a generic kinetic model for CMS hydrolysis with relative molarities at pH = 5.0 (Figure 6B).
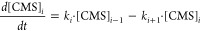
4

**Table 1:** Average Kinetic Rate Constants of the Hydrolysis of CMS at Three Concentrations of CMS Hydrolysis, Based on the HMS Profile Fitting with the Kinetic Model at pH = 5.0 (eq 5)

	kinetic rate constants (s^–1^) (×10^–3^)
*k*_1_: CMS (*i* = 0) → CMS^3–^ (*i* = 1)	1.0 ± 0.1
*k*_2_: CMS^3–^ (*i* = 1) → CMS^–^ (*i* = 2)	0.5 ± 0.2
*k*_3_: CMS^–^ (*i* = 2) → CMS^+^ (*i* = 3)	0.15 ± 0.01
*k*_4_: CMS^+^ (*i* = 3) → CMS^3+^ (*i* = 4)	0.4 ± 0.2
*k*_5_: CMS^3+^ (*i* = 4) → colistin (*i* = 5)	0.4 ± 0.2

in which *i* is the number of the compound starting at 0 and *k* is the first-order kinetic rate constant from CMS to colistin in five steps, chronologically named after every step in the reaction, starting from 1. The theoretical concentration of HMS that is formed can then be calculated by
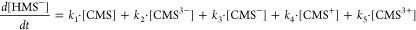
5

With the relation between the kinetic model and HMS formation (eq 5), we fitted the equation to the MS data from [HMS^–^] concentrations using least-squares fitting routines for three concentrations (Figure 6A). It was found that the hydrolysis profile of HMS is concentration-independent after normalization (inset in Figure 6A). To obtain well and unique fits for those three sets of MS data, five different (first order) kinetic rate constants (eq 5) were taken as input parameters—*k*_1_, *k*_2_, *k*_3_, *k*_4_, and *k*_5_. Trial and error of the coupling of several combinations of kinetic rate constants was imposed, yet proved to be ineffective, leaving all kinetic rate constants as independent parameters. Since the formation kinetics of HMS were found to be concentration-independent, we averaged the three kinetic model fits (separate fits in Table S4) and presented them in Table 1. Subsequently, the average kinetic rate constants were applied to eq 5, resulting in a generic kinetic model for CMS hydrolysis with relative molarities at pH = 5.0 (Figure 6B).

Based on the obtained kinetic rate constants, the first step of CMS hydrolysis is the quickest, after which the other steps have significantly lower rate constants (Table 1). However, note that the relative molarities do not directly translate into nominal concentrations. The conversion from CMS^–^ to CMS^+^ (k_3_) seems to be the rate-limiting step, while the other steps (*k*_2_, *k*_4_, and *k*_5_) all have similar, but due to high standard deviation, indistinctive kinetic rate constants. The combination of these kinetic rate constants results in a comparatively large molar fraction of CMS^–^ among other hydrolysis products and an onset of several hours in colistin formation (Figure 1B).

### 3.3 Relating the Slow Motion Complex Coacervation Process to the Hydrolysis Kinetics

**Figure 7:**
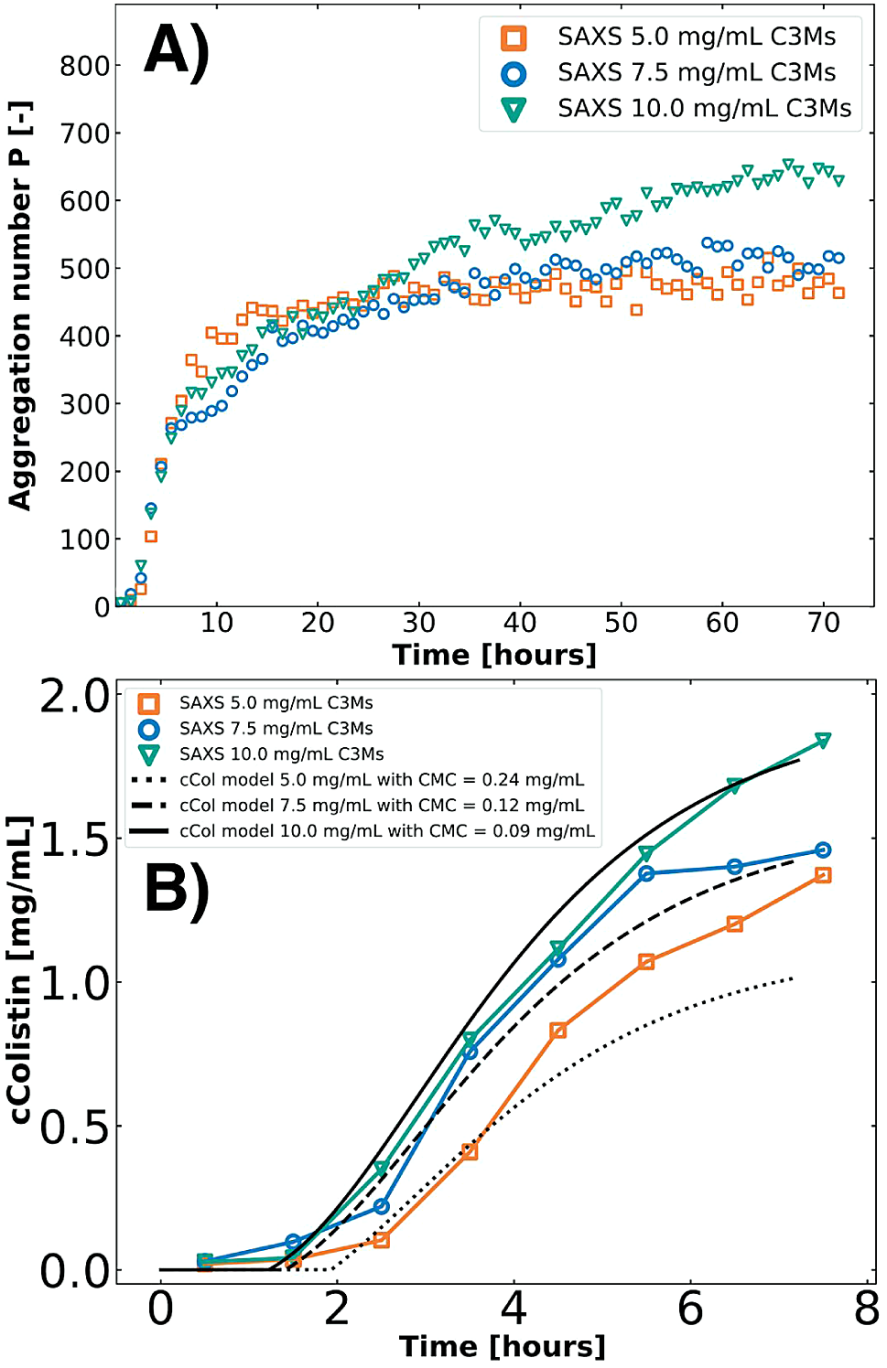
Aggregation number *P*, calculated by Guinier analysis, over time for total concentrations of 5.0 mg/mL (orange squares), 7.5 mg/mL (blue circles), and 10.0 mg/mL (green triangles) (A). Combining MS kinetic profile fitting (total concentrations: 5.0 mg/mL (orange squares), 7.5 mg/mL (blue circles), and 10.0 mg/mL (green triangles)) and their corresponding modeled formation profiles for colistin for total concentrations of 5.0 mg/mL (dotted line), 7.5 mg/mL (dashed), and 10.0 mg/mL (solid line) (B). Concentrations of colistin were imposed to match the lag time of the SAXS caused by micellization.

Although it is clear from the MS data that the hydrolysis process itself is concentration-independent, the micellization process will depend on the concentration. First, we expect the micellization to only take place above a CMC, i.e., the amount of hydrolyzed CMS needs to exceed a certain threshold value. Second, conditions will differ for the hydrolysis concentrations, based on several factors like local concentration variance^64^ and deviations in entropic gain due to counterion release, but also based on the number of charges that affect the electrostatic interactions.^65,66^ Consequently, micellization can start at different times for different concentrations of colistin and can potentially be different from the more generic CMC of the final system, which was found to be 0.29 ± 0.06 mg/mL at pH = 5.0 (Figure S3). By Guinier analysis of three different concentrations of CMS C3Ms (Figure S4), we calculated the growth of the aggregation number over time (Figure 7A), which we converted to a colistin concentration, assuming that 100% hydrolyzed CMS (colistin) is the only cationic species to interact. When we compared this colistin concentration from SAXS data to the kinetic concentration modeling of colistin, we observed an additional lag time in the aggregation number data, indicating differing “CMCs of formation” for all concentrations. Therefore, we imposed multiple CMCs for the micellization, corresponding to the observed lag times (Figure 7B).

From the Guinier analysis, we observe similar trends in aggregation numbers with higher concentrations leading to slightly higher aggregation numbers, potentially caused by slight aggregation (Figures 7A and S4). The trends in the colistin concentrations from SAXS and kinetic modeling, considering different CMCs of formation, show similar behavior although they do not directly match (Figure 7B). Nevertheless, the data indicate that fully hydrolyzed CMS (colistin) is necessary for effective complexation. The lower CMCs of formation at higher concentrations can be potentially due to higher ionic strength, and/or a slight change in pH. An additional factor can be the lower local concentration variance at higher concentrations and lower number of charges, causing a faster start of micellization.^65,66^

**Figure 8:**
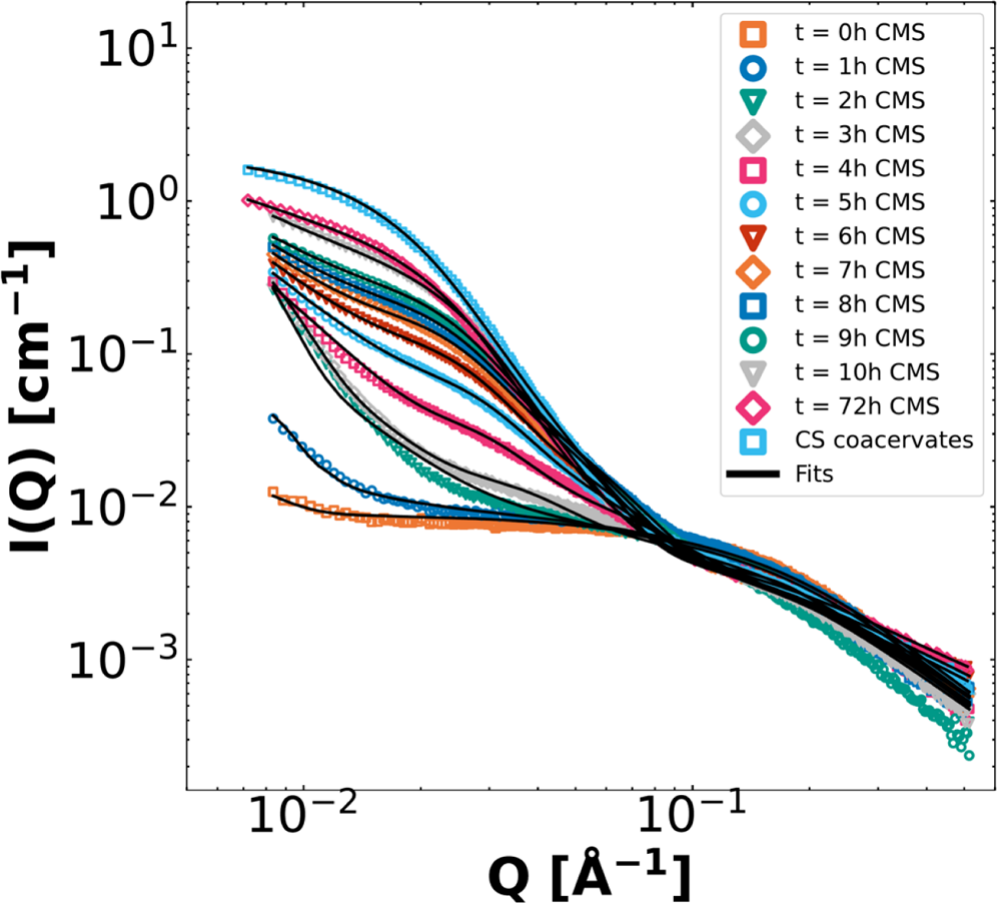
Aggregation number *P*, calculated by Guinier analysis, over time for total concentrations of 5.0 mg/mL (orange squares), 7.5 mg/mL (blue circles), and 10.0 mg/mL (green triangles) (A). Combining MS kinetic profile fitting (total concentrations 5.0 mg/mL (orange squares), 7.5 mg/mL (blue circles), and 10.0 mg/mL (green triangles)) and their corresponding modeled formation profiles for colistin for total concentrations 5.0 mg/mL (dotted line), 7.5 mg/mL (dashed), and 10.0 mg/mL (solid line) (B). Concentrations of colistin were imposed to match the lag time of the SAXS caused by micellization.

To gain more detailed insights into the micellization and growth kinetics of C3Ms and to elucidate a potential coacervation mechanism, we measured the first 10 h of complex coacervation with synchrotron SAXS at pH = 5.0 (BM29, ESRF) for one concentration (total 5.0 mg/mL). We employed the CMS/CS complex coacervate model, including the concentration of colistin from the kinetic model as the input, to fit the data to quantify the size and composition at several time steps in the C3M formation. In Figure 8, we present the SAXS patterns, including fits (all fit parameters can be found in Table S5), while in Table 2, the most relevant fitting parameters for all measurements are depicted.

**Table 2:** Most Relevant Fitting Parameters^a^; pH = 5.0 Complex Coacervation of Hydrolyzed CMS with PEO-b-PMAA for *t* = 0 to 10 h with a 1 h Step Interval, Final State (*t* = 72 h), and C3Ms at pH = 5.0 (CS)

*t* (h)	*P* (−) (×10^2^)	*R*_tot_ (nm)	*c*_col_^b^	*f*_col_ (−)	*M*_w_ (MDa) (×10^6^)	*f*_w_ (−)
0.0	0.0 ± 0.0	1.2 ± 0.4	0.00	0.00	0.00 ± 0.00	0.6 ± 0.1
1.0	0.4 ± 0.1	3.4 ± 0.1	0.02	0.00	0.06 ± 0.01	0.56 ± 0.07
2.0	0.5 ± 0.1	6.0 ± 0.2	0.21	0.00	0.09 ± 0.02	0.88 ± 0.02
3.0	0.7 ± 0.1	6.7 ± 0.3	0.56	0.00	0.12 ± 0.01	0.88 ± 0.02
4.0	1.5 ± 0.0	8.8 ± 0.2	0.92	0.00	0.25 ± 0.00	0.89 ± 0.01
5.0	2.1 ± 0.0	9.0 ± 0.0	1.20	0.00	0.35 ± 0.00	0.85 ± 0.01
6.0	2.9 ± 0.1	9.6 ± 0.1	1.39	0.00	0.50 ± 0.02	0.83 ± 0.01
7.0	2.9 ± 0.0	9.8 ± 0.0	1.51	0.07	0.48 ± 0.00	0.84 ± 0.01
8.0	2.9 ± 0.1	10.3 ± 0.1	1.61	0.09	0.48 ± 0.02	0.86 ± 0.02
9.0	3.3 ± 0.1	10.4 ± 0.2	1.67	0.10	0.54 ± 0.02	0.85 ± 0.02
10	4.3 ± 0.1	10.6 ± 0.1	1.71	0.12	0.71 ± 0.02	0.82 ± 0.02
72	4.8 ± 0.0	11.0 ± 0.1	1.83	0.14	0.81 ± 0.01	0.81 ± 0.01
CS	6.8 ± 0.1	13.5 ± 0.0	2.89	0.45^c^	1.13 ± 0.02	0.88 ± 0.01

a Standard deviation in the fits was determined by manual parameter changing, maximally allowing a 10% increase in *Χ*^2^, which we used as a parameter to ensure least-squares fitting.

b *c*_col_ is the concentration (mg/mL) of colistin from CMS, calculated based on the concentration of colistin at certain time points in the kinetic model (Figure 6B).

c Not all CS is complexed by the PEO-b-PMAA because of reduced polymer charge at pH = 5.0, and fits are made with a free fraction of CS of 45%, which was a fitting parameter.

Within the first hour of CMS hydrolysis, some small polymer-colistin complexes already started to form, seemingly mostly due to aggregation, shown by the low volume fraction of water (Figure 8, Table 2) and large cluster formation, visible by the upturn at low Q (Table S5). A small fraction of aggregates started to form after one and two hours, possibly containing a minor fraction of micellar structures, after which the CMC is crossed around the two-hour mark. Between 3 and 10 h, the hydrolysis of CMS in combination with PEO-b-PMAA results in C3Ms growing from 6.7 to 10 nm, while the volume fraction of water, *f*_w_, and therefore the molecular density roughly remain constant (Table 2). At the end of this period, the formed colistin is not all directly complexed, as shown by the increased free fraction of colistin, and starts stabilizing the C3Ms, reducing the aggregation at low *Q*. After 10 h, the *P* and radius start reaching a plateau in hydrolysis, thus resulting in a low increase of radius and molecular weight between 10 and 72 h. Based on these slow kinetic coacervation measurements, a three-step complex coacervation formation seems most appropriate: i) formation of small aggregates below CMC; ii) micelle formation, and fusion/fission caused by unbalanced charge resulting in growth; and iii) the saturation and stabilization of the micelles, potentially caused by polymer/colistin exchange.^1,2,49,60,67,68^ The CMS hydrolysis product and CS C3Ms are in the same order of composition and size but nevertheless slightly different. The CS C3Ms are bigger and have a higher water content, possibly related to the excess of colistin adding cationic ionic strength to the mixture.^32^ Another factor resulting in a difference is the preparation method. Slow coacervation will most likely lead to more but smaller micelles, whereas fast coacervation kinetics lead to fewer but bigger micelles.^40,49,50,68,69^

## 4 Conclusion

In this work, we developed two complementary approaches to quantify CMS hydrolysis fundamentally. By the addition of the partly anionic block copolymer PEO-b-PMAA to CMS, hydrolyzing into the cationic colistin, we were able to follow the hydrolysis of CMS through complex coacervation using SAXS. The formed C3Ms were analyzed with a CMS/CS complex coacervate SAXS model for fuzzy-surface spheres to determine the size, structure, and composition. Additionally, by measuring the hydrolysis profile of hydroxy methanesulfonate (HMS) with MS, the hydrolysis product released at every step in the reaction, we elucidated a simplified first-order kinetic model for CMS hydrolysis. We prove a fast initial cleavage of HMS, followed by slower hydrolysis, with the rate-limiting step involving charge switching. Combining the kinetic model with the complex coacervate data proved the necessity of the hydrolysis end-product colistin for complex coacervation and a multistep formation kinetic process, showing aggregation, growth, and slight rearrangements. Our findings contribute to a better understanding of CMS hydrolysis, including intermediate products, using relatively simple newly designed methodologies. Aside from their fundamental importance in unraveling the kinetics of clinically relevant antibiotic prodrugs, our findings also provide crucial insights into the kinetic pathways of complex electrostatically driven self-assembling systems. Complex coacervation was utilized not only to create drug delivery vehicles but also as a detection method for the hydrolysis of prodrugs. This dual application could potentially be relevant for other charged (pro)drugs, enhancing delivery and detection capabilities across a broader spectrum of pharmaceutical compounds. Moreover, the insight may be useful for formulations involving fragile cargo, such as therapeutic proteins/peptides, and enzymes where complexation may protect against premature deactivation and degradation. These findings could thus be instrumental in improving the design of pharmaceutical drug formulations and enhancing their efficacy.

## Supporting Information Available

SAXS model fitting, MS model fitting, pH dependency of CMS/CS C3Ms with SAXS, effect of PEO-b-PMAA on the hydrolysis, the simplified hydrolysis pattern of CMS, full kinetic rate constants, CMC determination, concentration dependency, and full fitting parameters (PDF)

## Abbreviations

AMP: antimicrobial peptides
C3Ms: complex coacervate core micelles
CMC: critical micelle concentration
CMS: colistin methanesulfonate
CS: colistin sulfate
HMS: hydroxy methanesulfonate
MS: Mass spectrometry
PEO-b-PMAA: poly(ethylene oxide)-b-poly(methacrylic acid)
PDI: polydispersity index
SAXS: small-angle X-ray scattering

## References

[ref1] Bergen P. J.; Li J.; Rayner C. R.; Nation R. L. Colistin Methanesulfonate Is an Inactive Prodrug of Colistin against Pseudomonas Aeruginosa. Antimicrob. Agents Chemother. 2006, 50 (6), 1953–1958. 10.1128/AAC.00035-06. 16723551 PMC1479097

[ref2] Li J.; Milne R. W.; Nation R. L.; Turnidge J. D.; Coulthard K. Stability of Colistin and Colistin Methanesulfonate in Aqueous Media and Plasma as Determined by High-Performance Liquid Chromatography. Antimicrob. Agents Chemother. 2003, 47 (4), 1364–1370. 10.1128/AAC.47.4.1364-1370.2003. 12654671 PMC152538

[ref3] Tacconelli E.; Carrara E.; Savoldi A.; Harbarth S.; Mendelson M.; Monnet D. L.; Pulcini C.; Kahlmeter G.; Kluytmans J.; Carmeli Y.; Ouellette M.; Outterson K.; Patel J.; Cavaleri M.; Cox E. M.; Houchens C. R.; Grayson M. L.; Hansen P.; Singh N.; Theuretzbacher U.; Magrini N.; Aboderin A. O.; Al-Abri S. S.; Awang Jalil N.; Benzonana N.; Bhattacharya S.; Brink A. J.; Burkert F. R.; Cars O.; Cornaglia G.; Dyar O. J.; Friedrich A. W.; Gales A. C.; Gandra S.; Giske C. G.; Goff D. A.; Goossens H.; Gottlieb T.; Guzman Blanco M.; Hryniewicz W.; Kattula D.; Jinks T.; Kanj S. S.; Kerr L.; Kieny M.-P.; Kim Y. S.; Kozlov R. S.; Labarca J.; Laxminarayan R.; Leder K.; Leibovici L.; Levy-Hara G.; Littman J.; Malhotra-Kumar S.; Manchanda V.; Moja L.; Ndoye B.; Pan A.; Paterson D. L.; Paul M.; Qiu H.; Ramon-Pardo P.; Rodríguez-Baño J.; Sanguinetti M.; Sengupta S.; Sharland M.; Si-Mehand M.; Silver L. L.; Song W.; Steinbakk M.; Thomsen J.; Thwaites G. E.; van der Meer J. W.; Van Kinh N.; Vega S.; Villegas M. V.; Wechsler-Fördös A.; Wertheim H. F. L.; Wesangula E.; Woodford N.; Yilmaz F. O.; Zorzet A. Discovery, Research, and Development of New Antibiotics: The WHO Priority List of Antibiotic-Resistant Bacteria and Tuberculosis. Lancet Infect. Dis. 2018, 18 (3), 318–327. 10.1016/S1473-3099(17)30753-3. 29276051

[ref4] Moretta A.; Scieuzo C.; Petrone A. M.; Salvia R.; Manniello M. D.; Franco A.; Lucchetti D.; Vassallo A.; Vogel H.; Sgambato A.; Falabella P. Antimicrobial Peptides: A New Hope in Biomedical and Pharmaceutical Fields. Front. Cell. Infect. Microbiol. 2021, 11, 668632 10.3389/fcimb.2021.668632. 34195099 PMC8238046

[ref5] Haney E. F.; Mansour S. C.; Hancock R. E. W. Antimicrobial Peptides: An Introduction. In Antimicrobial Peptides: Methods and Protocols, Hansen P. R.; ed. Springer; New York, NY, 2017; pp. 3 22. 10.1007/978-1-4939-6737-7_1. 28013493

[ref6] Browne K.; Chakraborty S.; Chen R.; Willcox M. D.; Black D. S.; Walsh W. R.; Kumar N. A New Era of Antibiotics: The Clinical Potential of Antimicrobial Peptides. Int. J. Mol. Sci. 2020, 21 (19), 7047 10.3390/ijms21197047. 32987946 PMC7582481

[ref7] Mahlapuu M.; Björn C.; Ekblom J. Antimicrobial Peptides as Therapeutic Agents: Opportunities and Challenges. Crit. Rev. Biotechnol. 2020, 40 (7), 978–992. 10.1080/07388551.2020.1796576. 32781848

[ref8] Nation R. L.; Li J. Colistin in the 21st Century. Curr. Opin. Infect. Dis. 2009, 22 (6), 535 10.1097/QCO.0b013e328332e672. 19797945 PMC2869076

[ref9] Huan Y.; Kong Q.; Mou H.; Yi H. Antimicrobial Peptides: Classification, Design, Application and Research Progress in Multiple Fields. Front. Microbiol. 2020, 11, 582779 10.3389/fmicb.2020.582779. 33178164 PMC7596191

[ref10] Falagas M. E.; Kasiakou S. K.; Saravolatz L. D. Colistin: The Revival of Polymyxins for the Management of Multidrug-Resistant Gram-Negative Bacterial Infections. Clin. Infect. Dis. 2005, 40 (9), 1333–1341. 10.1086/429323. 15825037

[ref11] Biswas S.; Brunel J.-M.; Dubus J.-C.; Reynaud-Gaubert M.; Rolain J.-M. Colistin: An Update on the Antibiotic of the 21st Century. Expert Rev. Anti-Infect. Ther. 2012, 10 (8), 917–934. 10.1586/eri.12.78. 23030331

[ref12] Dagla I.; Tsarbopoulos A.; Gikas E. Colistimethate Acidic Hydrolysis Revisited: Arrhenius Equation Modeling Using UPLC-QToF MS. Molecules 2021, 26 (2), 447 10.3390/molecules26020447. 33467022 PMC7830259

[ref13] Zabidi M. S.; Abu Bakar R.; Musa N.; Wan Yusuf W. N. Analytical Methodologies for Measuring Colistin Levels in Pharmacokinetic Studies. J. Liq. Chromatogr. Relat. Technol. 2020, 43 (15–16), 671–686. 10.1080/10826076.2020.1783291.

[ref14] Barnett M.; Bushby S. R. M.; Wilkinson S. Sodium Sulphomethyl Derivatives of Polymyxins. Br. J. Pharmacol. Chemother. 1964, 23 (3), 552–574. 10.1111/j.1476-5381.1964.tb01610.x. 14256814 PMC1704012

[ref15] Beveridge E. G.; Martin A. J. Sodium Sulphomethyl Derivatives of Polymyxins. Br. J. Pharmacol. Chemother. 1967, 29 (2), 125–135. 10.1111/j.1476-5381.1967.tb01946.x. 4292405 PMC1557203

[ref16] Zavascki A. P.; Nation R. L. Nephrotoxicity of Polymyxins: Is There Any Difference between Colistimethate and Polymyxin B?. Antimicrob. Agents Chemother. 2017, 61 (3), e02319–16 10.1128/aac.02319-16. 27993859 PMC5328560

[ref17] Kim K.-Y.; Kim B.-H.; Kwack W. G.; Kwon H.-J.; Cho S.-H.; Kim C.-W. Simple and Robust LC–MS/MS Method for Quantification of Colistin Methanesulfonate and Colistin in Human Plasma for Therapeutic Drug Monitoring. J. Pharm. Biomed. Anal. 2023, 236, 115734 10.1016/j.jpba.2023.115734. 37776629

[ref18] He H.; Li J.-C.; Nation R. L.; Jacob J.; Chen G.; Lee H. J.; Tsuji B. T.; Thompson P. E.; Roberts K.; Velkov T.; Li J. Pharmacokinetics of Four Different Brands of Colistimethate and Formed Colistin in Rats. J. Antimicrob. Chemother. 2013, 68 (10), 2311–2317. 10.1093/jac/dkt207. 23749953 PMC3772743

[ref19] Barco S.; Castagnola E.; Mesini A.; Tripodi G.; Cangemi G. Potential Pitfalls in LC-MS/MS Quantification of Colistin for Therapeutic Drug Monitoring of Patients Treated with Colistimethate. J. Pharm. Biomed. Anal. 2019, 170, 193–195. 10.1016/j.jpba.2019.03.023. 30928894

[ref20] Cizmarova I.; Parrak V.; Secnik P. Jr.; Secnik P.; Sopko L.; Nemergutova K.; Kovac A.; Mikus P.; Piestansky J. A Simple and Green Capillary Electrophoresis-Mass Spectrometry Method for Therapeutic Drug Monitoring of Colistin in Clinical Plasma Samples. Heliyon 2023, 9 (12), E23111 10.1016/j.heliyon.2023.e23111. 38076102 PMC10703858

[ref21] Bihan K.; Lu Q.; Enjalbert M.; Apparuit M.; Langeron O.; Rouby J.-J.; Funck-Brentano C.; Zahr N. Determination of Colistin and Colistimethate Levels in Human Plasma and Urine by High-Performance Liquid Chromatography–Tandem Mass Spectrometry. Ther. Drug Monit. 2016, 38 (6), 796–803. 10.1097/FTD.0000000000000345. 27684296

[ref22] Jansson B.; Karvanen M.; Cars O.; Plachouras D.; Friberg L. E. Quantitative Analysis of Colistin A and Colistin B in Plasma and Culture Medium Using a Simple Precipitation Step Followed by LC/MS/MS. J. Pharm. Biomed. Anal. 2009, 49 (3), 760–767. 10.1016/j.jpba.2008.12.016. 19157746

[ref23] Mizuyachi K.; Hara K.; Wakamatsu A.; Nohda S.; Hirama T. Safety and Pharmacokinetic Evaluation of Intravenous Colistin Methanesulfonate Sodium in Japanese Healthy Male Subjects. Curr. Med. Res. Opin. 2011, 27 (12), 2261–2270. 10.1185/03007995.2011.626557. 21995648

[ref24] Qi B.; Gijsen M.; Van Brantegem P.; De Vocht T.; Deferm N.; Abza G. B.; Nauwelaerts N.; Wauters J.; Spriet I.; Annaert P. Quantitative Determination of Colistin A/B and Colistin Methanesulfonate in Biological Samples Using Hydrophilic Interaction Chromatography Tandem Mass Spectrometry. Drug Test. Anal. 2020, 12 (8), 1183–1195. 10.1002/dta.2812. 32336034

[ref25] Dagla I.; Tsarbopoulos A.; Gikas E. A Novel Validated Injectable Colistimethate Sodium Analysis Combining Advanced Chemometrics and Design of Experiments. Molecules 2021, 26 (6), 1546 10.3390/molecules26061546. 33799846 PMC8000333

[ref26] Metcalf A. P.; Hardaker L. E. A.; Hatley R. H. M. A Simple Method for Assaying Colistimethate Sodium in Pharmaceutical Aerosol Samples Using High Performance Liquid Chromatography. J. Pharm. Biomed. Anal. 2017, 142, 15–18. 10.1016/j.jpba.2017.04.021. 28477450

[ref27] Wallace S. J.; Li J.; Rayner Craig R.; Coulthard K.; Nation R. L. Stability of Colistin Methanesulfonate in Pharmaceutical Products and Solutions for Administration to Patients. Antimicrob. Agents Chemother. 2008, 52 (9), 3047–3051. 10.1128/aac.00103-08. 18606838 PMC2533443

[ref28] Mohamed A. F.; Cars O.; Friberg L. E. A Pharmacokinetic/Pharmacodynamic Model Developed for the Effect of Colistin on Pseudomonas Aeruginosa in Vitro with Evaluation of Population Pharmacokinetic Variability on Simulated Bacterial Killing. J. Antimicrob. Chemother. 2014, 69 (5), 1350–1361. 10.1093/jac/dkt520. 24474432

[ref29] Li J.; Turnidge J.; Milne R.; Nation R. L.; Coulthard K. In Vitro Pharmacodynamic Properties of Colistin and Colistin Methanesulfonate against Pseudomonas aeruginosaIsolates from Patients with Cystic Fibrosis. Antimicrob. Agents Chemother. 2001, 45 (3), 781–785. 10.1128/AAC.45.3.781-785.2001. 11181360 PMC90373

[ref30] Bozkurt-Guzel Ç.; Gerceker A. In Vitro Pharmacodynamic Properties of Colistin Methanesulfonate and Amikacin against Pseudomonas Aeruginosa. Indian J. Med. Microbiol. 2012, 30 (1), 34–38. 10.4103/0255-0857.93020. 22361758

[ref31] Zhu Y.; Monsel A.; Roberts J. A.; Pontikis K.; Mimoz O.; Rello J.; Qu J.; Rouby J.-J. on behalf of the European Investigator Network for Nebulized Antibiotics in Ventilator-Associated Pneumonia (ENAVAP). Nebulized Colistin in Ventilator-Associated Pneumonia and Tracheobronchitis: Historical Background, Pharmacokinetics and Perspectives. Microorganisms 2021, 9 (6), 1154 10.3390/microorganisms9061154. 34072189 PMC8227626

[ref32] Vogelaar T. D.; Agger A. E.; Reseland J. E.; Linke D.; Jenssen H.; Lund R. Crafting Stable Antibiotic Nanoparticles via Complex Coacervation of Colistin with Block Copolymers. Biomacromolecules 2024, 25, 4267 10.1021/acs.biomac.4c00337. 38886154 PMC11238337

[ref33] Amann M.; Diget J. S.; Lyngso̷ J.; Pedersen J. S.; Narayanan T.; Lund R. Kinetic Pathways for Polyelectrolyte Coacervate Micelle Formation Revealed by Time-Resolved Synchrotron SAXS. Macromolecules 2019, 52 (21), 8227–8237. 10.1021/acs.macromol.9b01072.

[ref34] Heo T.-Y.; Kim S.; Chen L.; Sokolova A.; Lee S.; Choi S.-H. Molecular Exchange Kinetics in Complex Coacervate Core Micelles: Role of Associative Interaction. ACS Macro Lett. 2021, 10 (9), 1138–1144. 10.1021/acsmacrolett.1c00482. 35549078

[ref35] Blocher McTigue W. C.; Perry S. L. Protein Encapsulation Using Complex Coacervates: What Nature Has to Teach Us. Small 2020, 16 (27), 1907671 10.1002/smll.201907671. 32363758

[ref36] Shah S.; Leon L. Structural Dynamics, Phase Behavior, and Applications of Polyelectrolyte Complex Micelles. Curr. Opin. Colloid Interface Sci. 2021, 53, 101424 10.1016/j.cocis.2021.101424.

[ref37] Nolles A.; Hooiveld E.; Westphal A. H.; van Berkel W. J. H.; Kleijn J. M.; Borst J. W. FRET Reveals the Formation and Exchange Dynamics of Protein-Containing Complex Coacervate Core Micelles. Langmuir 2018, 34 (40), 12083–12092. 10.1021/acs.langmuir.8b01272. 30212214 PMC6209312

[ref38] van der Kooij H. M.; Spruijt E.; Voets I. K.; Fokkink R.; Cohen Stuart M. A.; van der Gucht J. On the Stability and Morphology of Complex Coacervate Core Micelles: From Spherical to Wormlike Micelles. Langmuir 2012, 28 (40), 14180–14191. 10.1021/la303211b. 22978707

[ref39] Bos I.; Brink E.; Michels L.; Sprakel J. DNA Dynamics in Complex Coacervate Droplets and Micelles. Soft Matter 2022, 18 (10), 2012–2027. 10.1039/D1SM01787J. 35191449 PMC8905490

[ref40] Voets I. K.; de Keizer A.; Cohen Stuart M. A. Complex Coacervate Core Micelles. Adv. Colloid Interface Sci. 2009, 147–148, 300–318. 10.1016/j.cis.2008.09.012. 19038373

[ref41] Magana J. R.; Sproncken C. C. M.; Voets I. K. On Complex Coacervate Core Micelles: Structure-Function Perspectives. Polymers 2020, 12 (9), 1953 10.3390/polym12091953. 32872312 PMC7565781

[ref42] Rumyantsev A. M.; Jackson N. E.; de Pablo J. J. Polyelectrolyte Complex Coacervates: Recent Developments and New Frontiers. Annu. Rev. Condens. Matter. Phys. 2021, 12 (1), 155–176. 10.1146/annurev-conmatphys-042020-113457.

[ref43] Blocher W. C.; Perry S. L. Complex Coacervate-Based Materials for Biomedicine. Wiley Interdiscip. Rev.: Nanomed. Nanobiotechnol. 2017, 9 (4), e1442 10.1002/wnan.1442. 27813275

[ref44] Voets I. K.; Moll P. M.; Aqil A.; Jérôme C.; Detrembleur C.; Waard P. D.; Keizer A. D.; Stuart M. A. C. Temperature Responsive Complex Coacervate Core Micelles With a PEO and PNIPAAm Corona. J. Phys. Chem. B 2008, 112 (35), 10833–10840. 10.1021/jp8014832. 18698810

[ref45] Hofs B.; Brzozowska A.; de Keizer A.; Norde W.; Cohen Stuart M. A. Reduction of Protein Adsorption to a Solid Surface by a Coating Composed of Polymeric Micelles with a Glass-like Core. J. Colloid Interface Sci. 2008, 325 (2), 309–315. 10.1016/j.jcis.2008.06.006. 18589433

[ref46] Da Vela S.; Svergun D. I. Methods, Development and Applications of Small-Angle X-Ray Scattering to Characterize Biological Macromolecules in Solution. Curr. Res. Struct. Biol. 2020, 2, 164–170. 10.1016/j.crstbi.2020.08.004. 34235476 PMC8244429

[ref47] Bos I.; Timmerman M.; Sprakel J. FRET-Based Determination of the Exchange Dynamics of Complex Coacervate Core Micelles. Macromolecules 2021, 54 (1), 398–411. 10.1021/acs.macromol.0c02387. 33456072 PMC7808214

[ref48] Liu X.; Haddou M.; Grillo I.; Mana Z.; Chapel J.-P.; Schatz C. Early Stage Kinetics of Polyelectrolyte Complex Coacervation Monitored through Stopped-Flow Light Scattering. Soft Matter 2016, 12 (44), 9030–9038. 10.1039/C6SM01979J. 27748777

[ref49] Takahashi R.; Narayanan T.; Sato T. Growth Kinetics of Polyelectrolyte Complexes Formed from Oppositely-Charged Homopolymers Studied by Time-Resolved Ultra-Small-Angle X-Ray Scattering. J. Phys. Chem. Lett. 2017, 8 (4), 737–741. 10.1021/acs.jpclett.6b02957. 28121154

[ref50] McTigue W. C. B.; Voke E.; Chang L.-W.; Perry S. L. The Benefit of Poor Mixing: Kinetics of Coacervation. Phys. Chem. Chem. Phys. 2020, 22 (36), 20643–20657. 10.1039/D0CP03224G. 32895678

[ref51] Heuser T.; Weyandt E.; Walther A. Biocatalytic Feedback-Driven Temporal Programming of Self-Regulating Peptide Hydrogels. Angew. Chem., Int. Ed. 2015, 54 (45), 13258–13262. 10.1002/anie.201505013. 26249239

[ref52] Heuser T.; Steppert A.-K.; Zhu B.; Walther A. Generic Concept to Program the Time Domain of Self-Assemblies with a Self-Regulation Mechanism. Nano Lett. 2015, 15 (4), 2213–2219. 10.1021/nl5039506. 25393204

[ref53] Panzarasa G.; Osypova A.; Sicher A.; Bruinink A.; Dufresne E. R. Controlled Formation of Chitosan Particles by a Clock Reaction. Soft Matter 2018, 14 (31), 6415–6418. 10.1039/C8SM01060A. 30062339

[ref54] Tully M. D.; Kieffer J.; Brennich M. E.; Cohen Aberdam R.; Florial J. B.; Hutin S.; Oscarsson M.; Beteva A.; Popov A.; Moussaoui D.; Theveneau P.; Papp G.; Gigmes J.; Cipriani F.; McCarthy A.; Zubieta C.; Mueller-Dieckmann C.; Leonard G.; Pernot P. BioSAXS at European Synchrotron Radiation Facility – Extremely Brilliant Source: BM29 with an Upgraded Source, Detector, Robot, Sample Environment. J. Synchrotron Radiat. 2023, 30 (1), 258–266. 10.1107/S1600577522011286. 36601945 PMC9814054

[ref55] Berndt I.; Pedersen J. S.; Lindner P.; Richtering W. Influence of Shell Thickness and Cross-Link Density on the Structure of Temperature-Sensitive Poly-N-Isopropylacrylamide–Poly-N-Isopropylmethacrylamide Core–Shell Microgels Investigated by Small-Angle Neutron Scattering. Langmuir 2006, 22 (1), 459–468. 10.1021/la052463u. 16378460

[ref56] Berndt I.; Pedersen J. S.; Richtering W. Temperature-Sensitive Core–Shell Microgel Particles with Dense Shell. Angew. Chem. 2006, 118 (11), 1769–1773. 10.1002/ange.200503888. 16470901

[ref57] Pedersen J. S. Structure Factors Effects in Small-Angle Scattering from Block Copolymer Micelles and Star Polymers. J. Chem. Phys. 2001, 114 (6), 2839–2846. 10.1063/1.1339221.

[ref58] Pedersen J. S.; Svaneborg C. Scattering from Block Copolymer Micelles. Curr. Opin. Colloid Interface Sci. 2002, 7 (3), 158–166. 10.1016/S1359-0294(02)00044-4.

[ref59] Burchard W.; Kajiwara K. The Statistics of Stiff Chain Molecules I. The Particle Scattering Factor. Proc. R. Soc. Lond. Math. Phys. Sci. 1970, 316 (1525), 185–199. 10.1098/rspa.1970.0074.

[ref60] Lund R.; Willner L.; Richter D. Kinetics of Block Copolymer Micelles Studied by Small-Angle Scattering Methods, In Controlled polymerization and polymeric structures: Flow microreactor polymerization, micelles kinetics, polypeptide ordering, light emitting nanostructures; Abe A.; Lee K.-S.; Leibler L.; Kobayashi S.; Eds.; Springer, Cham, 2013, pp. 51 158. 10.1007/12_2012_204.

[ref61] Debye P. Molecular-Weight Determination by Light Scattering. J. Phys. Colloid Chem. 1947, 51 (1), 18–32. 10.1021/j150451a002. 20286386

[ref62] Fang Y. N.; Rumyantsev A. M.; Neitzel A. E.; Liang H.; Heller W. T.; Nealey P. F.; Tirrell M. V.; de Pablo J. J. Scattering Evidence of Positional Charge Correlations in Polyelectrolyte Complexes. Proc. Natl. Acad. Sci. 2023, 120 (32), e2302151120 10.1073/pnas.2302151120. 37523553 PMC10410704

[ref63] Tian B.; Liu S.; Lu W.; Jin L.; Li Q.; Shi Y.; Li C.; Wang Z.; Du Y. Construction of pH-Responsive and up-Conversion Luminescent NaYF4: Yb3+/Er3+@SiO2@PMAA Nanocomposite for Colon Targeted Drug Delivery. Sci. Rep. 2016, 6 (1), 21335 10.1038/srep21335. 26891778 PMC4759527

[ref64] Li X.; Fang Y.; Al-Assaf S.; Phillips G. O.; Yao X.; Zhang Y.; Zhao M.; Zhang K.; Jiang F. Complexation of Bovine Serum Albumin and Sugar Beet Pectin: Structural Transitions and Phase Diagram. Langmuir 2012, 28 (27), 10164–10176. 10.1021/la302063u. 22697399

[ref65] Kayitmazer A. B. Thermodynamics of Complex Coacervation. Adv. Colloid Interface Sci. 2017, 239, 169–177. 10.1016/j.cis.2016.07.006. 27497750

[ref66] Souza H. K. S.; Sousa A. M. M.; Gómez J.; Gonçalves M. P. Complexation of WPI and Microwave-Assisted Extracted Agars with Different Physicochemical Properties. Carbohydr. Polym. 2012, 89 (4), 1073–1080. 10.1016/j.carbpol.2012.03.065. 24750916

[ref67] Wu H.; Ting J. M.; Yu B.; Jackson N. E.; Meng S.; de Pablo J. J.; Tirrell M. V. Spatiotemporal Formation and Growth Kinetics of Polyelectrolyte Complex Micelles with Millisecond Resolution. ACS Macro Lett. 2020, 9 (11), 1674–1680. 10.1021/acsmacrolett.0c00543. 35617069

[ref68] Thanh N. T. K.; Maclean N.; Mahiddine S. Mechanisms of Nucleation and Growth of Nanoparticles in Solution. Chem. Rev. 2014, 114 (15), 7610–7630. 10.1021/cr400544s. 25003956

[ref69] Zhang P.; Wang Z.-G. Supernatant Phase in Polyelectrolyte Complex Coacervation: Cluster Formation, Binodal, and Nucleation. Macromolecules 2022, 55 (10), 3910–3923. 10.1021/acs.macromol.2c00340.

