# Supplementary material for: Coacervation in Slow Motion: Kinetics of Complex Micelle Formation Induced by the Hydrolysis of an Antibiotic Prodrug
Source: Mol Pharm. 2024 Jul 16;21(8):4157–68. doi: 10.1021/acs.molpharmaceut.4c00579 (PMC11304390; doi:10.1021/acs.molpharmaceut.4c00579)
Supplement: Supplementary file 1 — mp4c00579_si_001.pdf [file mp4c00579_si_001.pdf]

Supporting Information for

Coacervation in Slow Motion: Kinetics of Complex Micelle Formation Induced by the Hydrolysis of an Antibiotic Prodrug

*Thomas D. Vogelaar[1], Szymon M. Szostak[1], and Reidar Lund[1,2]**

[1]Department of Chemistry, University of Oslo, P.O. Box 1033 Blindern, NO-0315 Oslo, Norway

[2]Hylleraas Centre for Quantum Molecular Sciences, University of Oslo, NO-0315 Oslo, Norway

Table of Contents

1. Detailed model fitting procedures
    1.1. SAXS model fitting
        1.1.1. Complex coacervate scattering
        1.1.2. Free component scattering
        1.1.3. Internal structure scattering
        1.1.4. CMS scattering
        1.1.5. Fitting strategies and output
    1.2. MS model fitting
2. Results and Discussion
    2.1. pH dependence
    2.2. Kinetically arrested state in hydrolysis
    2.3. Measuring hydrolysis kinetics via SAXS and MS
    2.4. Critical micelle concentration (CMC)
    2.5. Full spectrum slow motion kinetics fitting
3. References

1. Detailed model fitting procedures

1.1. SAXS model fitting

To fit the SAXS data, to resolve the size, composition, and structure of the different phases of complex coacervate formation, in parallel to the Guinier analysis a tailored CMS/CS complex coacervate model was used, which was large based on the model used in Vogelaar *et al.* (2024).[1] We refer to that work for the mass balance contributions and assumptions upon which this CMS/CS complex coacervate model is built. The model is based on several assumptions. Firstly, CMS and sulfomethylated derivatives are not able to interact with PEO-b-PMAA and form complexes. This assumption is made based on the MS kinetic modeling, described in this work. Secondly, to form complexes, we need fully hydrolyzed CMS: colistin. Lastly, other assumptions are that colistin will be incorporated in the complex coacervate structures upon formation until saturation is observed in the SAXS data, also that the maximum hydrolysis of CMS is dependent on pH, and that CMS and its sulfomethylated derivatives can be described by ordinary CMS scattering, even though partial hydrolysis has taken place. The total scattering curve, used for modeling in this paper is the following (Eq. S1):

$$I(Q) = \varphi \cdot f_{Coa} \left(f_{clu} \cdot S(Q)_{cluster} \cdot I_{Coa}(Q) + (1 - f_{clu}) \cdot I_{Coa}(Q) + \frac{blob\ (Q)}{V_{Coa}}\right) + \varphi \left(f_{Poly} \cdot I_{Poly,free}(Q) \cdot f_{mix} + f_{Col} \cdot I_{Col,free}(Q) \cdot (1 - f_{mix})\right) + f_{Coa} \cdot S(Q)_{internal} + \varphi_{CMS} \cdot I_{CMS}(Q) \quad \text{(S1)}$$

Where φ is the volume fraction. f_{Coa} is the fraction of coacervates, f_{clu} is the fraction that is forming clusters, V_{Coa} is the volume of one complex coacervate, f_{Poly} is the free fraction of polymer, f_{Col} is the free fraction of colistin, f_{mix} is the molar fraction of polymer in the aqueous phase surrounding the complex coacervates, and ϕ_{CMS} is the volume fraction of CMS. Compared to the previous publication,[1] the CMS/CS complex coacervate model is very similar, apart from the fact that we introduced a scattering component from CMS, that the free fraction of colistin is fixed, and the concentration of colistin as an input parameter that is based on the fitted MS data.

The scattering model in Equation 1, consists of three scattering contributions: complex coacervate scattering, free component scattering, and an internal structure factor from the internal spacing inside the polyelectrolyte core. The first part of the scattering model describes the complex coacervate scattering and includes a cluster structure factor if required. The second part describes the free component scattering from the polymer and colistin. The third part is an additional scattering contribution from a pseudo-structure factor describing positional correlations between oppositely charged electrostatic blobs.[2] The last part describes the additional scattering from CMS and their sulfomethylated derivatives after partial hydrolysis. The first two, and the last contributions are based on the scattering function of centrosymmetric particles (Eq. S2).

$$I(Q) = n \cdot \Delta\rho^2 \cdot {V_{tot}}^2 \cdot P(Q) \cdot S(Q) \quad \text{(S2)}$$

In which n is the number density of scatterers, the $\Delta\rho$ the scattering length density (SLD) contrast, the V_{tot} is the total volume of the coacervates/free chains, $P(Q)$ is the form factor of

the coacervates/free chains and *S(Q)* is the structure factor of the coacervates/free chains. Generally, the n can be written as follows (Eq. S3).

$$n = \frac{\varphi}{V_{dry}} \qquad \text{(S3)}$$

Where V_{dry} is the volume of the coacervates/free chains excluding the water.

1.1.1. Complex coacervate scattering

First, we start with the explanation of the complex coacervate scattering. Equation S2 was used to describe the scattering of complex coacervates excluding the volume fraction ϕ. For the complex coacervates, the scattering amplitude, and therefore the form factor, of a fuzzy-core particle (P(Q) = $A_{core}(Q)^2$) can be described as follows (Eq. S4-S8).[3,4]

$$F_0 = \left(\frac{R}{\sigma^2} + \frac{1}{\sigma}\right) \cdot \frac{\cos(Q \cdot (R+\sigma))}{Q^4} - \frac{3 \cdot \sin(Q \cdot (R+\sigma))}{Q^5 \cdot \sigma^2} + \left(\frac{R}{\sigma^2} - \frac{1}{\sigma}\right) \cdot \frac{\cos(Q(R-\sigma))}{Q^4} \qquad \text{(S4)}$$

$$F_1 = \frac{-3 \cdot \sin(Q \cdot (R+\sigma)) + 6 \cdot \sin(Q * R)}{Q^5 \cdot \sigma^2} - \frac{2 \cdot R \cdot \cos(Q \cdot R)}{Q^4 \cdot \sigma^2} \qquad \text{(S5)}$$

$$V_n = \frac{R^3}{3} + \frac{R \cdot \sigma^2}{6} \qquad \text{(S6)}$$

$$A_{core}(Q) = \frac{F_0 + F_1}{V_n} \qquad \text{(S7)}$$

$$I_{coa}(Q) = \frac{\Delta\rho_{average}{}^2 \cdot P^2 \cdot V_{tot}{}^2 \cdot A_{core}(Q)^2}{V_{Coa}} \qquad \text{(S8)}$$

Where I_{Coa}(Q) is the scattering contribution from the complex coacervates, *P* is the aggregation number, the number of molecules that make up one micelle, and $A_{core}(Q)$ is the scattering amplitude of the fuzzy core. For the complex coacervates, if there is any cluster formation, a structure factor is needed to explain the scattering. The structure factor for clusters, $S(Q)_{cluster}$, is described by the following equations (Eq. S9-14).[5]

$$p = N_{clu} - floor(N_{clu}) \qquad \text{(S9)}$$

$$D = 2 \cdot R_{tot} \cdot D_{dist} \qquad \text{(S10)}$$

$$S_x = \frac{\sin(Q \cdot D)}{Q \cdot D} \qquad \text{(S11)}$$

$$S_n = \frac{2}{1-S_x} - 1 - \frac{2\left(1 - S_x{}^{floor(N_{clu})}\right) \cdot S_x}{floor(N_{clu}) \cdot (1-S_x)^2} \qquad \text{(S12)}$$

$$S_{n1} = \frac{2}{1-S_x} - 1 - \frac{2\left(1-S_x^{(floor(N_{clu})+1)}\right)\cdot S_x}{(floor(N_{clu})+1)\cdot(1-S_x)^2} \qquad \text{(S13)}$$

$$S(Q)_{cluster} = (1-p)\cdot S_n + p\cdot S_{n1} \qquad \text{(S14)}$$

In which N_{clu} is the number of clusters and D_{dist} is the distance correlation of the clusters. Apart from the structure factor, there is also a scattering contribution from the blob scattering from the polyelectrolytes in the core. The blob scattering is described in the following equation (Eq. S15).

$$blob\,(Q) = V_{tot}^2 \cdot P \cdot \Delta\rho_{average}^2 \cdot \frac{|f_{blob}|}{(1+Q^2\cdot\xi^2)} \qquad \text{(S15)}$$

In which the f_{blob} is the fraction of blob scattering, and ξ is the correlation length of the blobs. The complex coacervate scattering is highly dependent on the formation of colistin since all the colistin is quickly incorporated into the complex coacervates upon formation based on trial and error. The concentration of colistin is based on the kinetic model based on the MS data and scaled with the maximum hydrolyzable fraction of CMS from Figure 4.

1.1.2. Free component scattering

In the second part, we describe the free component scattering of polymer and colistin. To describe the free component scattering, we use a form factor instead of a scattering amplitude. In the free component scattering, the Debye form factor *P(Q)* for polymers and polyelectrolytes describes the scattering (Eq. S16).[6] The form factors are taken for each component and implemented in their scattering equations. (Eq. S17-S18).

$$P(Q)_{Debye} = \frac{2(e^{-Q^2\cdot R_g{}^2} - 1 + Q^2\cdot R_g{}^2)}{(Q^2\cdot R_g{}^2)^2} \qquad \text{(S16)}$$

$$I(Q)_{Poly,free} = \frac{(\frac{M_{PMAA}}{d_{PMAA}} + \frac{M_{PEO}}{d_{PEO}})\cdot \Delta\rho_{Poly}{}^2 \cdot P(Q)_{Debye,\,Poly}}{N_A} \qquad \text{(S17)}$$

$$I(Q)_{Col,free} = \frac{(\frac{M_{Col0}}{d_{Col}})\cdot \Delta\rho_{Col}{}^2 \cdot P(Q)_{Debye,\,Col}}{N_A} \qquad \text{(S18)}$$

Where N_A is Avogadro's number. For the added components, to obtain the SLD and R_g for the form factor, the contributions were first fitted by the Debye model using least-squares fit routines. The Debye model in the general form can be written as follows (Eq. S19).

$$I(Q) = \varphi \cdot \frac{\frac{M}{d}\cdot \Delta\rho^2 \cdot P(Q)_{Debye}}{N_A} \qquad \text{(S19)}$$

The Debye model was used, in which the R_g and $\Delta\rho$ were fitted for the free fractions of colistin and polymer, after which they were used in the CMS/CS complex coacervate model.

1.1.3. Internal structure scattering

In the complex coacervates, we see an internal structure from the bump in scattering around 0.22 $Å^{-1}$, which can be described by positional charges in polyelectrolyte complexes. To add that to the CMS/CS complex coacervate model, we have taken a scattering contribution as a pseudo-structure factor $S(Q)_{internal}$ on top of the existing scattering contributions, which was written as follows (Eq. S20).

$$S(Q)_{internal} = \frac{|C| \cdot e^{-(Q-Q_{local})^2}}{W \cdot Q_{local} \cdot \sqrt{2\pi} \cdot 2 \cdot (W \cdot Q_{local})^2} \qquad \text{(S20)}$$

In which $S(Q)_{internal}$ is the pseudo-structure factor, C fractal scattering, Q_{local} is the position of the structure peak, and W is the relative width of the local Q. These three parameters were fitted in the CMS/CS complex coacervate model.

1.1.4. CMS scattering

As mentioned earlier, the addition of CMS scattering includes both CMS, but also its sulfomethylated derivates, with the assumption that all components scatter similarly to CMS scattering that was measured by itself, and fitted with the Debye model, described in S19 to obtain the scattering length density and R_g, that were used in the CMS/CS complex coacervate model. The scattering contribution of CMS was described as follows (Eq. S21).

$$I(Q)_{CMS} = \frac{(\frac{M_{CMS}}{d_{CMS}}) \cdot \Delta\rho_{CMS}{}^2 \cdot P(Q)_{Debye,\,CMS}}{N_A} \qquad \text{(S21)}$$

The volume fraction of CMS was calculated, based on the input colistin concentration, which was an input parameter from MS modeling, assuming mass conservation in the system, scaled by molecular weight differences and volume differences and taking into account the maximum hydrolyzable fraction from Figure 4.

1.1.5. Fitting strategies and output

For the fit analysis, we utilized least-squares fit routines, maintaining flexibility in the following parameters: the overall aggregation number (P), the core radius encompassing its density distribution and polydispersity (R_{in}, σ_{in}, PDI), the unbound fraction of colistin (f_{Col}), and, when applicable, in the presence of cluster formation, the number of complexes in a cluster (N_{clu}) and the distance between complexes (D_{dist}). These parameters primarily characterize the low and intermediate Q range. After achieving an initial fit for the low and intermediate Q regions, an additional fitting process was employed to accurately represent the data at high Q by considering the scattering from the blobs themselves. Furthermore, we adjusted the blob scattering parameters, including the blob fraction (f_{blob}) and the blob correlation length (ξ). Lastly, to account for blob charge correlations (internal structure), we conducted fitting for the relative width at high Q (W), internal scattering location (Q_{local}), and fractal scattering (C). Ultimately, all parameters underwent simultaneous fitting to the data.

Based on the fits we were mostly interested in the total radius of the coacervate structures (R_{tot}), its PDI, and aggregation number P. Additionally, based on the fit parameters and outcomes, we could calculate the molecular weight of the coacervate structures and the water volume fraction with the following equations (Eq. S22-S23).

$$M_w = P \cdot (f_{mix0} \cdot (M_{PMAA} + M_{PEO}) + (1 - f_{mix0}) \cdot M_{Col0}) \qquad \text{(S22)}$$

$$f_w = 1 - \frac{P \cdot V_{tot}}{\frac{4}{3}\pi \cdot ({R_{in}}^3 + \frac{R_{in} \cdot {\sigma_{in}}^2}{2})} \qquad \text{(S23)}$$

Where f_{mix0} is the theoretical molar fraction of polymer if charge matching conditions apply. To determine the standard deviation in the fitting parameters, for the fitting parameters presented in this work, we changed the fitting parameter dependency, maximally allowing a 10% increase in X^2, which we used as a parameter to assure least-squares fitting. This was only applied to the data fitted in the table, to show the quality of the fits.

1.2. MS model fitting

To fit the data, based on the hydrolysis profile of methanesulfonate groups in the form of hydroxy methanesulfonate (HMS), a set of six differential equations was used, describing all the present different charged species in the hydrolysis of CMS over time (Eq. S24-S29) and calculated the formation of HMS over time (Eq. S30).

$$\frac{d[CMS]}{dt} = -\, k_1 \cdot [CMS] \qquad \text{(S24)}$$

$$\frac{d[CMS^{3-}]}{dt} = k_1 \cdot \; [CMS] - k_2 \cdot [CMS^{3-}] \qquad \text{(S25)}$$

$$\frac{d[CMS^{-}]}{dt} = k_2 \cdot \; [CMS^{3-}] - k_3 \cdot [CMS^{-}] \qquad \text{(S26)}$$

$$\frac{d[CMS^{+}]}{dt} = k_3 \cdot \; [CMS^{-}] - k_4 \cdot [CMS^{+}] \qquad \text{(S27)}$$

$$\frac{d[CMS^{3+}]}{dt} = k_4 \cdot \; [CMS^{+}] - k_5 \cdot [CMS^{3+}] \qquad \text{(S28)}$$

$$\frac{d[CS]}{dt} = k_5 \cdot \; [CMS^{3+}] \qquad \text{(S29)}$$

$$\frac{d[HMS^{-}]}{dt} = k_1 \cdot [CMS] + k_2 \cdot [CMS^{3-}] + k_3 \cdot [CMS^{-}] + k_4 \cdot [CMS^{+}] + \; k_5 \cdot [CMS^{3+}] \qquad \text{(S30)}$$

We fitted the MS data on the formation of HMS with the model equations described above, using least-square fit routines, in which k_1, k_2, k_3, k_4, and k_5 were fitting parameters and kept free. To include concentration and pH dependency, we fitted three HMS patterns at pH = 5.0 at three concentrations (1.8 mg/mL CMS, 3.6 mg/mL CMS, and 7.2 mg/mL CMS) and one HMS pattern at pH = 7.4 (at 3.6 mg/mL CMS) and obtained different kinetic rate constants for all the curves. To determine the concentrations of species of CMS hydrolysis over time, we averaged the kinetic rate constants of the different concentrations and inserted them as input in S24-S29, resulting in a kinetic model. To convert to colistin concentrations, we took the same ratios of HMS formation, measured with MS, as these are scalable, when the CMS fraction not taking part in the hydrolysis, is considered inert, resulting in relative molarities.

2. Results and Discussion

2.1. pH dependence

Table S1: Fitting parameters from the final state of CMS + PEO-b-PMAA after t = 72h at 5.0 mg/mL total concentration. Obtained from the CMS/CS complex coacervate model.

**Model parameters**[a]	pH = 5.0	pH = 6.0	pH = 7.0	pH = 7.4	pH = 8.0	pH = 8.7
M_{Col} (Da)	1406	1406	1406	1406	1406	1406
M_{CMS0} (Da)	1636	1636	1636	1636	1636	1636
$\Delta\rho_{Col}$ (cm^{-2})	$1.24{\cdot}10^{11}$	$1.24{\cdot}10^{11}$	$1.24{\cdot}10^{11}$	$1.24{\cdot}10^{11}$	$1.24{\cdot}10^{11}$	$1.24{\cdot}10^{11}$
$\Delta\rho_{PMAA}$ (cm^{-2})	$1.09{\cdot}10^{11}$	$1.09{\cdot}10^{11}$	$1.09{\cdot}10^{11}$	$1.09{\cdot}10^{11}$	$1.09{\cdot}10^{11}$	$1.09{\cdot}10^{11}$
$\Delta\rho_{PEO}$ (cm^{-2})	$1.11{\cdot}10^{11}$	$1.11{\cdot}10^{11}$	$1.11{\cdot}10^{11}$	$1.11{\cdot}10^{11}$	$1.11{\cdot}10^{11}$	$1.11{\cdot}10^{11}$
$\Delta\rho_{Core}$ (cm^{-2})	$1.24{\cdot}10^{11}$	$1.24{\cdot}10^{11}$	$1.24{\cdot}10^{11}$	$1.24{\cdot}10^{11}$	$1.24{\cdot}10^{11}$	$1.24{\cdot}10^{11}$
$\Delta\rho_{CMS}$ (cm^{-2})	$1.31{\cdot}10^{11}$	$1.35{\cdot}10^{11}$	$1.32{\cdot}10^{11}$	$1.35{\cdot}10^{11}$	$1.43{\cdot}10^{11}$	$1.45{\cdot}10^{11}$
$\Delta\rho_{Solvent}$ (cm^{-2})	$9.43{\cdot}10^{10}$	$9.43{\cdot}10^{10}$	$9.43{\cdot}10^{10}$	$9.43{\cdot}10^{10}$	$9.43{\cdot}10^{10}$	$9.43{\cdot}10^{10}$
f_{mix0} (-)	0.093	0.093	0.093	0.093	0.093	0.093
c_{Poly} (mg/mL)	1.40	1.40	1.40	1.40	1.40	1.40
c_{Col} (mg/mL)	1.83	1.83	0.55	0.49	0.26	0.17
P (-)	$4.8{\cdot}10^{2}$	$5.3{\cdot}10^{1}$	$2.0{\cdot}10^{2}$	$2.3{\cdot}10^{2}$	$2.8{\cdot}10^{2}$	$2.4{\cdot}10^{2}$
σ_{in} (nm)	0.0	0.0	0.0	0.0	0.0	0.0
σ_{out} (nm)	0.0	0.0	0.0	0.0	0.0	0.0
R_{in} (nm)	11.0	4.3	3.6	3.6	3.3	2.8
R_{tot} (nm)	11.0	4.3	3.6	3.6	3.3	2.8
f_{clu} (-)	0.07	0.48	0.05	0.05	0.00	1.00
D_{dist} (-)	0.99	1.70	1.09	0.97	0.13	1.00
f_{coa} (-)	0.72	0.72	0.42	0.39	0.23	0.16
$R_{g,Col}$ (nm)	0.64	0.64	0.64	0.64	0.64	0.64
$R_{g,Poly}$ (nm)	2.43	2.43	2.43	2.43	2.43	2.43
$R_{g,CMS}$ (nm)[b]	0.89	0.98	1.15	1.28	1.31	1.30
C (-)	$3{\cdot}10^{-3}$	$5{\cdot}10^{-3}$	$5{\cdot}10^{-3}$	$2{\cdot}10^{-4}$	$3{\cdot}10^{-4}$	$1{\cdot}10^{-4}$
f_{blob} (-)	0.83	0.38	0.26	1.00	0.99	0.19
ξ (-)	4.0	1.4	0.4	0.3	0.0	1.5
W (-)	2.00	0.13	0.13	0.14	0.21	0.14
Q_{local} ($Å^{-1}$)	0.13	0.22	0.23	0.22	0.23	0.23
M_{Col0} (Da)	1165	1165	1165	1165	1165	1165
f_{Col} (-)	0.14	0.14	0.00	0.00	0.00	0.00
f_{Poly} (-)	0.46	0.46	0.81	0.83	0.91	0.94
f_{mix} (-)	0.35	0.35	1.00	1.00	1.00	1.00
M_w (Da)	$8.1{\cdot}10^{5}$	$8.8{\cdot}10^{4}$	$3.4{\cdot}10^{5}$	$3.9{\cdot}10^{5}$	$4.6{\cdot}10^{5}$	$4.0{\cdot}10^{5}$
c_{ColC3M} (mg/mL)	1.57	1.57	0.55	0.49	0.26	0.17
c_{CMS} (mg/mL)	1.33	1.33	2.92	2.99	3.28	3.39
f_w (-)	0.81	0.66	0.00	0.00	0.00	0.00
PDI (-)	0.25	0.19	0.19	0.19	0.20	0.20

[a]All fitting parameters, without standard deviation, resulted in a minimized X^2, the parameter we used to assure least-square fitting.
[b]The radius of gyration from CMS is based on fitting CMS data at several pH values with the Debye model.

Table S2: Fitting parameters of CS coacervates at an equivalent concentration of 5.0 mg/mL of CMS + PEO-b-PMAA, measured at different pH values. Obtained from the CMS/CS complex coacervate model.

**Model parameters**[a]	pH = 5.0	pH = 6.0	pH = 7.0	pH = 7.4	pH = 8.0	pH = 8.7
M_{Col} (Da)	1406	1406	1406	1406	1406	1406
$\Delta\rho_{Col}$ (cm^{-2})	$1.24\cdot10^{11}$	$1.24\cdot10^{11}$	$1.24\cdot10^{11}$	$1.24\cdot10^{11}$	$1.24\cdot10^{11}$	$1.24\cdot10^{11}$
$\Delta\rho_{PMAA}$ (cm^{-2})	$1.09\cdot10^{11}$	$1.09\cdot10^{11}$	$1.09\cdot10^{11}$	$1.09\cdot10^{11}$	$1.09\cdot10^{11}$	$1.09\cdot10^{11}$
$\Delta\rho_{PEO}$ (cm^{-2})	$1.11\cdot10^{11}$	$1.11\cdot10^{11}$	$1.11\cdot10^{11}$	$1.11\cdot10^{11}$	$1.11\cdot10^{11}$	$1.11\cdot10^{11}$
$\Delta\rho_{Core}$ (cm^{-2})	$1.24\cdot10^{11}$	$1.24\cdot10^{11}$	$1.24\cdot10^{11}$	$1.24\cdot10^{11}$	$1.24\cdot10^{11}$	$1.24\cdot10^{11}$
$\Delta\rho_{Solvent}$ (cm^{-2})	$9.43\cdot10^{10}$	$9.43\cdot10^{10}$	$9.43\cdot10^{10}$	$9.43\cdot10^{10}$	$9.43\cdot10^{10}$	$9.43\cdot10^{10}$
f_{mix0} (-)	0.093	0.093	0.093	0.093	0.093	0.093
c_{Poly} (mg/mL)	1.40	1.40	1.40	1.40	1.40	1.40
c_{Col} (mg/mL)	2.89	2.89	2.89	2.89	2.89	2.89
P (-)	$6.8\cdot10^{2}$	$6.6\cdot10^{2}$	$1.1\cdot10^{3}$	$1.9\cdot10^{3}$	$1.6\cdot10^{3}$	$1.9\cdot10^{3}$
σ_{in} (nm)	0.0	0.0	0.0	0.0	0.0	0.0
σ_{out} (nm)	0.0	0.0	0.0	0.0	0.0	0.0
R_{in} (nm)	13.5	11.9	13.3	14.3	14.1	14.5
R_{tot} (nm)	13.5	11.9	13.3	14.3	14.1	14.5
f_{clu} (-)	0.00	0.01	0.20	0.00	0.01	0.00
D_{dist} (-)	1.00	0.31	1.38	1.00	0.27	1.00
f_{coa} (-)	0.55	0.55	0.72	0.86	0.89	0.97
$R_{g,Col}$ (nm)	0.64	0.64	0.64	0.64	0.64	0.64
$R_{g,Poly}$ (nm)	2.43	2.43	2.43	2.43	2.43	2.43
C (-)	$3\cdot10^{-4}$	$1\cdot10^{-4}$	$5\cdot10^{-5}$	$1\cdot10^{-4}$	$1\cdot10^{-4}$	$1\cdot10^{-4}$
f_{blob} (-)	0.31	0.10	0.26	0.17	0.19	0.19
ξ (-)	2.1	1.2	2.2	1.2	1.7	1.5
W (-)	0.39	0.16	0.09	0.14	0.13	0.14
Q_{local} (Å^{-1})	0.14	0.21	0.22	0.23	0.22	0.23
M_{Col0} (Da)	1165	1165	1165	1165	1165	1165
f_{Col} (-)	0.45	0.45	0.28	0.14	0.10	0.03
f_{Poly} (-)	0.45	0.45	0.28	0.14	0.10	0.03
f_{mix} (-)	0.09	0.09	0.09	0.09	0.09	0.09
M_w (Da)	$1.1\cdot10^{6}$	$1.1\cdot10^{6}$	$1.9\cdot10^{6}$	$3.2\cdot10^{6}$	$2.6\cdot10^{6}$	$3.1\cdot10^{6}$
c_{ColC3M} (mg/mL)	1.58	1.58	2.09	2.49	2.60	2.81
f_w (-)	0.88	0.83	0.79	0.71	0.76	0.73
PDI (-)	0.28	0.26	0.23	0.16	0.17	0.17

[a]All fitting parameters, without standard deviation, resulted in a minimized X^2, the parameter we used to assure least-square fitting.

2.2. Kinetically arrested state in hydrolysis

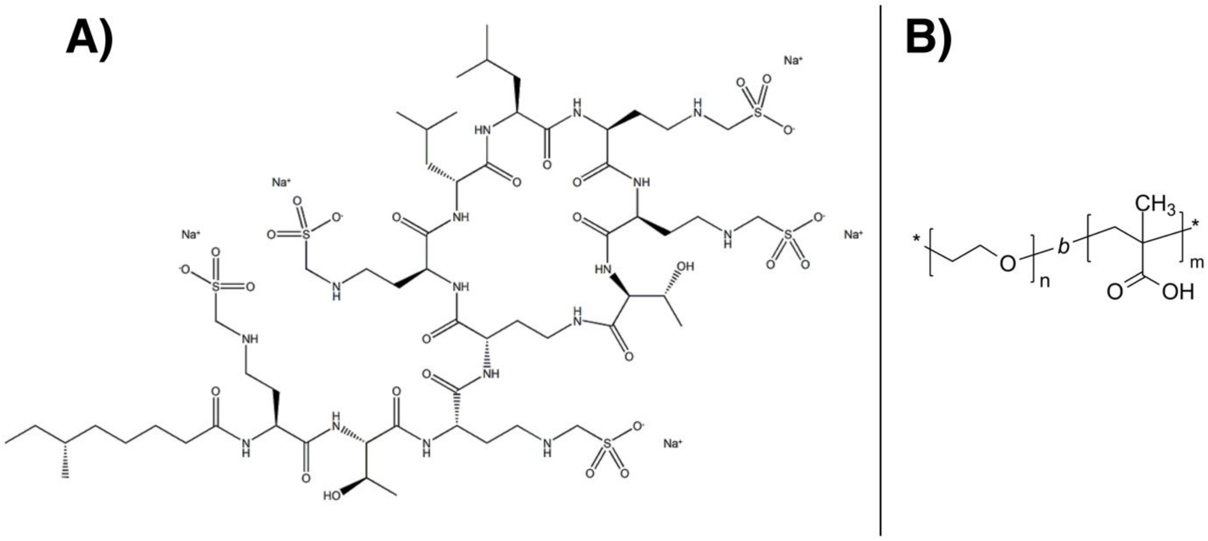

Figure S1: Structural formulas of sodium colistin methanesulfonate A (CMS) (A) and PEO_n-b-$PMAA_m$ (B). Structural formulas are adapted from Chemicalbook and Polymersource respectively. Sodium CMS consists of 75% of A and 25% of the B version, which is one carbon in the fatty acid tail shorter.

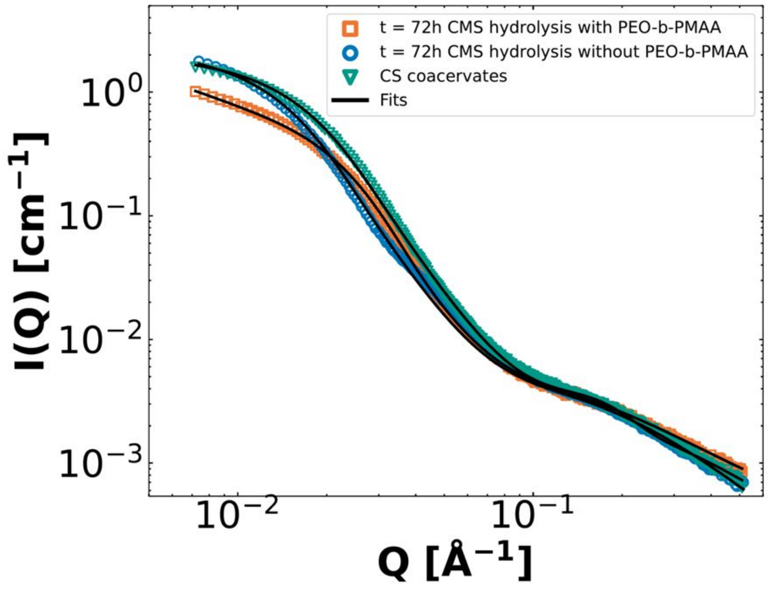

Figure S2: Hydrolysis of CMS at pH = 5.0 in the presence of PEO-b-PMAA (orange squares), or without PEO-b-PMAA, after which polymer is added to complex the formed colistin (blue circles). Comparison is made with CS coacervates in equivalent concentration and pH (green triangles).

Table S3: Fitting parameters of the hydrolysis of CMS at pH = 5.0 in the presence of PEO-b-PMAA, or without PEO-b-PMAA, after which polymer is added to complex the formed colistin. Comparison to CS coacervates. Obtained from the CMS/CS complex coacervate model.

**Model parameters**[a]	With PEO-b-PMAA	Without PEO-b-PMAA	CS coacervates
M_{Col} (Da)	1406	1406	1406

M_{CMS0} (Da)	1636	1636	-
$\Delta\rho_{Col}$ (cm^{-2})	$1.24\cdot10^{11}$	$1.24\cdot10^{11}$	$1.24\cdot10^{11}$
$\Delta\rho_{PMAA}$ (cm^{-2})	$1.09\cdot10^{11}$	$1.09\cdot10^{11}$	$1.09\cdot10^{11}$
$\Delta\rho_{PEO}$ (cm^{-2})	$1.11\cdot10^{11}$	$1.11\cdot10^{11}$	$1.11\cdot10^{11}$
$\Delta\rho_{Core}$ (cm^{-2})	$1.24\cdot10^{11}$	$1.24\cdot10^{11}$	$1.24\cdot10^{11}$
$\Delta\rho_{CMS}$ (cm^{-2})	$1.31\cdot10^{11}$	$1.31\cdot10^{11}$	-
$\Delta\rho_{Solvent}$ (cm^{-2})	$9.43\cdot10^{10}$	$9.43\cdot10^{10}$	$9.43\cdot10^{10}$
f_{mix0} (-)	0.093	0.093	0.093
c_{Poly} (mg/mL)	1.40	1.40	1.40
c_{Col} (mg/mL)	1.83	1.83	2.89
P (-)	$4.8\cdot10^{2}$	$1.3\cdot10^{3}$	$6.8\cdot10^{2}$
σ_{in} (nm)	0.0	0.0	0.0
σ_{out} (nm)	0.0	0.0	0.0
R_{in} (nm)	11.0	16.1	13.5
R_{tot} (nm)	11.0	16.1	13.5
f_{clu} (-)	0.07	0.00	0.00
D_{dist} (-)	0.99	1.00	1.00
f_{coa} (-)	0.72	0.72	0.54
$R_{g,Col}$ (nm)	0.64	0.64	0.64
$R_{g,Poly}$ (nm)	2.43	2.43	2.43
$R_{g,CMS}$ (nm)	0.89	0.89	-
C (-)	$3\cdot10^{-3}$	$4\cdot10^{-5}$	$3\cdot10^{-4}$
f_{blob} (-)	0.83	1.00	0.31
ξ (-)	4.0	4.0	2.1
W (-)	2.0	20	0.4
Q_{local} (Å^{-1})	0.13	0.56	0.14
M_{Col0} (Da)	1165	1165	1165
f_{Col} (-)	0.14	0.14	0.45
f_{Poly} (-)	0.46	0.46	0.45
f_{mix} (-)	0.35	0.35	0.09
M_w (Da)	$8.1\cdot10^{5}$	$2.1\cdot10^{6}$	$1.1\cdot10^{6}$
c_{ColC3M} (mg/mL)	1.57	1.57	1.58
c_{CMS} (mg/mL)	1.33	1.33	-
f_w (-)	0.81	0.84	0.88
PDI (-)	0.25	0.27	0.28

[a]All fitting parameters, without standard deviation, resulted in a minimized X^2, the parameter we used to assure least-square fitting.

2.3. Measuring hydrolysis kinetics via SAXS and MS

Table S4: Kinetic rate constants based on the CMS hydrolysis kinetic model at pH = 5.0 at three different concentrations of CMS hydrolysis.

Kinetic rate constants · 10^{-3}	1.8 mg/mL CMS	3.6 mg/mL CMS	7.2 mg/mL CMS
k1: CMS (i = 0) → CMS^{3-} (i = 1)	0.93	1.07	1.11
k2: CMS^{3-} (i = 1) → CMS^{-} (i = 2)	0.69	0.48	0.34
k3: CMS^{-} (i = 2) → CMS^{+} (i = 3)	0.14	0.14	0.16
k4: CMS^{+} (i = 3) → CMS^{3+} (i = 4)	0.28	0.31	0.58
k5: CMS^{3+} (i = 4) → Colistin (i = 5)	0.45	0.47	0.20
The lowest sum of squares for fit	$6.1\cdot10^{-4}$	$8.9\cdot10^{-5}$	$2.9\cdot10^{-4}$

2.4. Critical micelle concentration (CMC)

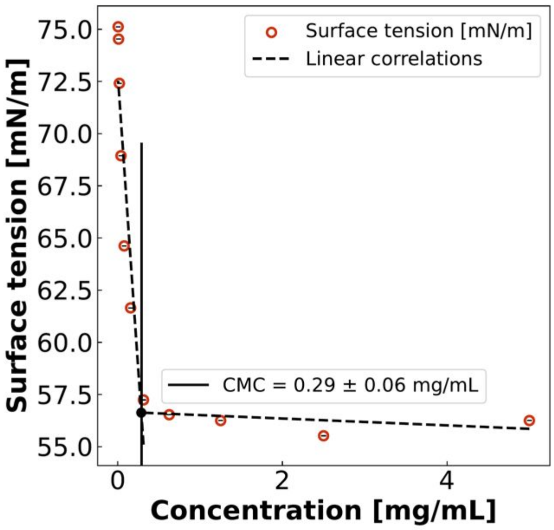

Figure S3: Surface tension measurements over a concentration range to determine the CMC of CMS hydrolyzed C3Ms prepared at a total concentration of 5.0 mg/mL. With the pendant drop method, the surface tension was determined at several concentrations, by which two regions could be determined: high slope and low slope. The intercept is the CMC of the coacervate complexes, which was determined to be 0.29 ± 0.06 mg/mL by linear regression of both lines.

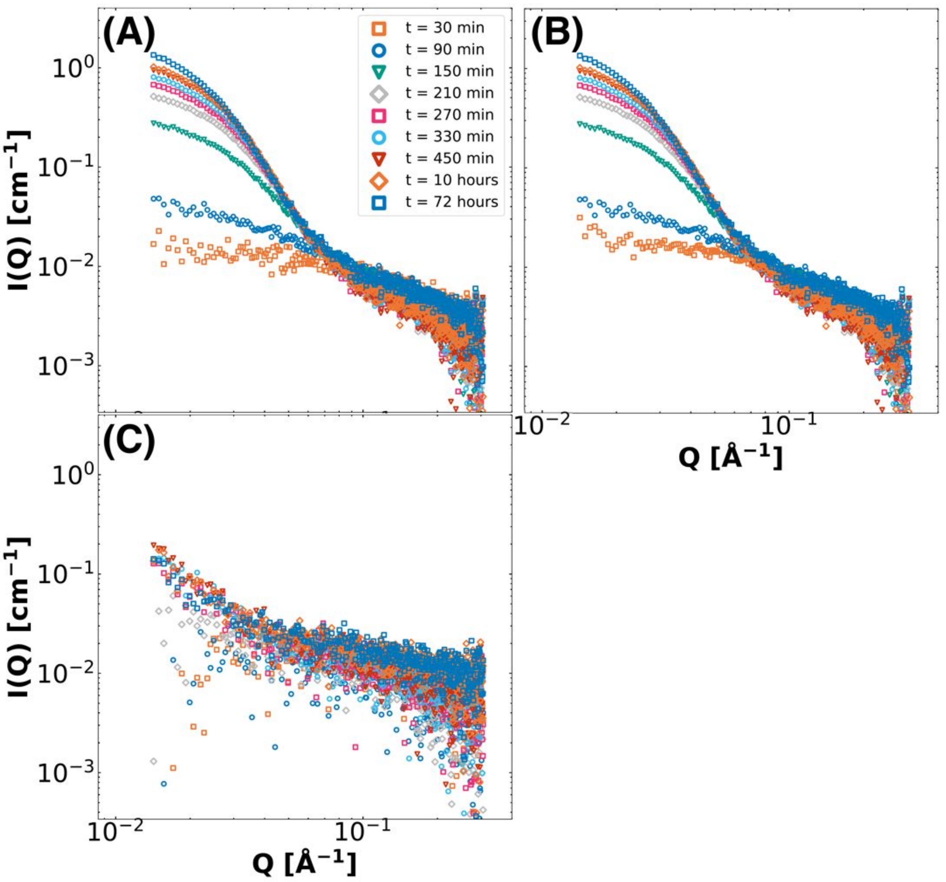

Figure S4: SAXS pattern representation of the coacervation process over time of CMS with PEO-b-PMAA at a total concentration of 10.0 mg/mL (A), 7.5 mg/mL (B), and 2.5 mg/mL (B) for 72 hours. The same graph window was used for all plots to visualize the changes in scattering intensity between the different pHs in the clearest manner.

2.5. Full spectrum slow motion kinetics fitting

Table S5: Fitting parameters of the hydrolysis of CMS at pH = 5.0 in the presence of PEO-b-PMAA over time, including a comparison to CS coacervates. Obtained from the CMS/CS complex coacervate model.

**Model parameters[a]**	**t = 0h**	**t = 1h**	**t = 2h**	**t = 3h**	**t = 4h**	**t = 5h**	**t = 6h**	**t = 7h**	**t = 8h**	**t = 9h**	**t = 10h**	**t = 72h**	**CS coacervates**
M_{Col} (Da)	1406	1406	1406	1406	1406	1406	1406	1406	1406	1406	1406	1406	1406
M_{CMS} (Da)	1636	1636	1636	1636	1636	1636	1636	1636	1636	1636	1636	1636	-
$\Delta\rho_{Col}$ (cm^{-2})	$1.24{\cdot}10^{11}$	$1.24{\cdot}10^{11}$	$1.24{\cdot}10^{11}$	$1.24{\cdot}10^{11}$	$1.24{\cdot}10^{11}$	$1.24{\cdot}10^{11}$	$1.24{\cdot}10^{11}$	$1.24{\cdot}10^{11}$	$1.24{\cdot}10^{11}$	$1.24{\cdot}10^{11}$	$1.24{\cdot}10^{11}$	$1.24{\cdot}10^{11}$	$1.24{\cdot}10^{11}$
$\Delta\rho_{PMAA}$ (cm^{-2})	$1.09{\cdot}10^{11}$	$1.09{\cdot}10^{11}$	$1.09{\cdot}10^{11}$	$1.09{\cdot}10^{11}$	$1.09{\cdot}10^{11}$	$1.09{\cdot}10^{11}$	$1.09{\cdot}10^{11}$	$1.09{\cdot}10^{11}$	$1.09{\cdot}10^{11}$	$1.09{\cdot}10^{11}$	$1.09{\cdot}10^{11}$	$1.09{\cdot}10^{11}$	$1.09{\cdot}10^{11}$
$\Delta\rho_{PEO}$ (cm^{-2})	$1.11{\cdot}10^{11}$	$1.11{\cdot}10^{11}$	$1.11{\cdot}10^{11}$	$1.11{\cdot}10^{11}$	$1.11{\cdot}10^{11}$	$1.11{\cdot}10^{11}$	$1.11{\cdot}10^{11}$	$1.11{\cdot}10^{11}$	$1.11{\cdot}10^{11}$	$1.11{\cdot}10^{11}$	$1.11{\cdot}10^{11}$	$1.11{\cdot}10^{11}$	$1.11{\cdot}10^{11}$
$\Delta\rho_{Core}$ (cm^{-2})	$1.24{\cdot}10^{11}$	$1.24{\cdot}10^{11}$	$1.24{\cdot}10^{11}$	$1.24{\cdot}10^{11}$	$1.24{\cdot}10^{11}$	$1.24{\cdot}10^{11}$	$1.24{\cdot}10^{11}$	$1.24{\cdot}10^{11}$	$1.24{\cdot}10^{11}$	$1.24{\cdot}10^{11}$	$1.24{\cdot}10^{11}$	$1.24{\cdot}10^{11}$	$1.24{\cdot}10^{11}$
$\Delta\rho_{CMS}$ (cm^{-2})	$1.31{\cdot}10^{11}$	$1.31{\cdot}10^{11}$	$1.31{\cdot}10^{11}$	$1.31{\cdot}10^{11}$	$1.31{\cdot}10^{11}$	$1.31{\cdot}10^{11}$	$1.31{\cdot}10^{11}$	$1.31{\cdot}10^{11}$	$1.31{\cdot}10^{11}$	$1.31{\cdot}10^{11}$	$1.31{\cdot}10^{11}$	$1.31{\cdot}10^{11}$	-
$\Delta\rho_{Solvent}$ (cm^{-2})	$9.43{\cdot}10^{10}$	$9.43{\cdot}10^{10}$	$9.43{\cdot}10^{10}$	$9.43{\cdot}10^{10}$	$9.43{\cdot}10^{10}$	$9.43{\cdot}10^{10}$	$9.43{\cdot}10^{10}$	$9.43{\cdot}10^{10}$	$9.43{\cdot}10^{10}$	$9.43{\cdot}10^{10}$	$9.43{\cdot}10^{10}$	$9.43{\cdot}10^{10}$	$9.43{\cdot}10^{10}$
f_{mix0} (-)	0.093	0.093	0.093	0.093	0.093	0.093	0.093	0.093	0.093	0.093	0.093	0.093	0.093
c_{Poly} (mg/mL)	1.40	1.40	1.40	1.40	1.40	1.40	1.40	1.40	1.40	1.40	1.40	1.40	1.40
c_{Col} (mg/mL)	0.00	0.02	0.21	0.56	0.92	1.20	1.39	1.51	1.61	1.67	1.71	1.83	2.89
P (-)	$0.0{\cdot}10^2$	$0.4{\cdot}10^2$	$0.5{\cdot}10^2$	$0.7{\cdot}10^2$	$1.5{\cdot}10^2$	$2.1{\cdot}10^2$	$2.9{\cdot}10^2$	$2.9{\cdot}10^2$	$2.9{\cdot}10^2$	$3.3{\cdot}10^2$	$4.3{\cdot}10^2$	$4.8{\cdot}10^2$	$6.8{\cdot}10^2$
σ_{in} (nm)	0.0	0.0	0.0	0.0	0.0	0.0	0.0	0.0	0.0	0.0	0.0	0.0	0.0
σ_{out} (nm)	0.0	0.0	0.0	0.0	0.0	0.0	0.0	0.0	0.0	0.0	0.0	0.0	0.0
R_{in} (nm)	1.2	3.4	6.1	6.7	8.8	9.0	9.6	9.8	10.3	10.4	10.6	11.0	13.5
R_{tot} (nm)	1.2	3.4	6.1	6.7	8.8	9.0	9.6	9.8	10.3	10.4	10.6	11.0	13.5
f_{clu} (-)	0.00	0.13	0.00	0.00	0.00	0.68	0.59	0.51	0.47	0.39	0.22	0.07	0.00
D_{dist} (-)	1.20	0.45	0.92	1.33	1.25	1.34	1.45	1.55	1.51	1.50	1.31	0.99	1.00
f_{coa} (-)	0.00	0.02	0.19	0.42	0.59	0.68	0.74	0.72	0.72	0.73	0.72	0.72	0.54
$R_{g,Col}$ (nm)	0.64	0.64	0.64	0.64	0.64	0.64	0.64	0.64	0.64	0.64	0.64	0.64	0.64
$R_{g,Poly}$ (nm)	2.43	2.43	2.43	2.43	2.43	2.43	2.43	2.43	2.43	2.43	2.43	2.43	2.43
$R_{g,CMS}$ (nm)	0.89	0.89	0.89	0.89	0.89	0.89	0.89	0.89	0.89	0.89	0.89	0.89	-
C (-)	0.03	0.03	0.10	0.01	$1{\cdot}10^{-3}$	$1{\cdot}10^{-3}$	$2{\cdot}10^{-3}$	$7{\cdot}10^{-4}$	$4{\cdot}10^{-3}$	$4{\cdot}10^{-3}$	$3{\cdot}10^{-3}$	$3{\cdot}10^{-3}$	$3{\cdot}10^{-4}$
f_{blob} (-)	0.10	1.00	0.01	0.03	0.10	0.97	1.00	1.00	1.00	1.00	1.00	0.83	0.31
ξ (-)	3.8	3.8	3.8	1.0	1.0	4.0	1.6	4.0	4.0	4.0	4.0	4.0	2.1
W (-)	1.2	1.2	4.0	1.2	4.0	4.0	4.0	0.5	2.4	1.2	3.5	2.0	0.4
Q_{local} (Å^{-1})	0.00	0.00	0.00	0.00	0.00	0.01	0.00	0.00	0.04	0.00	0.00	0.13	0.14
M_{Col0} (Da)	1165	1165	1165	1165	1165	1165	1165	1165	1165	1165	1165	1165	1165
f_{Col} (-)	0.00	0.00	0.00	0.00	0.00	0.00	0.00	0.07	0.09	0.10	0.12	0.14	0.45
f_{Poly} (-)	1.00	0.99	0.93	0.81	0.68	0.59	0.52	0.52	0.49	0.48	0.48	0.46	0.45
f_{mix} (-)	1.00	1.00	1.00	1.00	1.00	1.00	1.00	0.59	0.50	0.46	0.41	0.35	0.09
M_w (Da)	$0.0{\cdot}10^5$	$0.6{\cdot}10^5$	$0.9{\cdot}10^5$	$1.2{\cdot}10^5$	$2.5{\cdot}10^5$	$3.5{\cdot}10^5$	$4.9{\cdot}10^5$	$4.8{\cdot}10^5$	$4.8{\cdot}10^5$	$5.4{\cdot}10^5$	$7.1{\cdot}10^5$	$8.1{\cdot}10^5$	$11.3{\cdot}10^5$
c_{ColC3M} (mg/mL)	0.00	0.02	0.21	0.56	0.92	1.20	1.39	1.40	1.47	1.50	1.50	1.57	1.58
c_{CMS} (mg/mL)	3.61	3.58	3.34	2.91	2.46	2.11	1.88	1.73	1.73	1.60	1.47	1.33	-
f_w (-)	0.00	0.56	0.88	0.88	0.89	0.85	0.83	0.84	0.86	0.85	0.82	0.81	0.88
PDI (-)	0.23	0.23	0.24	0.23	0.22	0.23	0.24	0.24	0.24	0.24	0.24	0.25	0.28

[a]All fitting parameters, without standard deviation, resulted in a minimized X^2, the parameter we used to assure least-square fitting.

3. References

(1) Vogelaar, T. D.; Agger, A. E.; Reseland, J. E.; Linke, D.; Jenssen, H.; Lund, R. Crafting Stable Antibiotic Nanoparticles via Complex Coacervation of Colistin with Block Copolymers. *Biomacromolecules* **2024**. https://doi.org/10.1021/acs.biomac.4c00337.

(2) Fang, Y. N.; Rumyantsev, A. M.; Neitzel, A. E.; Liang, H.; Heller, W. T.; Nealey, P. F.; Tirrell, M. V.; de Pablo, J. J. Scattering Evidence of Positional Charge Correlations in Polyelectrolyte Complexes. *Proc. Natl. Acad. Sci.* **2023**, *120* (32), e2302151120. https://doi.org/10.1073/pnas.2302151120.

(3) Berndt, I.; Pedersen, J. S.; Lindner, P.; Richtering, W. Influence of Shell Thickness and Cross-Link Density on the Structure of Temperature-Sensitive Poly-N-Isopropylacrylamide−Poly-N-Isopropylmethacrylamide Core−Shell Microgels Investigated by Small-Angle Neutron Scattering. *Langmuir* **2006**, *22* (1), 459–468. https://doi.org/10.1021/la052463u.

(4) Berndt, I.; Pedersen, J. S.; Richtering, W. Temperature-Sensitive Core–Shell Microgel Particles with Dense Shell. *Angew. Chem.* **2006**, *118* (11), 1769–1773. https://doi.org/10.1002/ange.200503888.

(5) Burchard, W.; Kajiwara, K.; Whiffen, D. H. The Statistics of Stiff Chain Molecules I. The Particle Scattering Factor. *Proc. R. Soc. Lond. Math. Phys. Sci.* **1997**, *316* (1525), 185–199. https://doi.org/10.1098/rspa.1970.0074.

(6) Debye, P. Molecular-Weight Determination by Light Scattering. *J. Phys. Colloid Chem.* **1947**, *51* (1), 18–32. https://doi.org/10.1021/j150451a002.
